# Mechanistic Study on Copper- and Silver-Catalyzed Hydroboration of Internal Alkynes: A DFT Study

**DOI:** 10.1021/acsorginorgau.5c00004

**Published:** 2025-03-11

**Authors:** Ivanna G. R. Juliani Costa, Patrick R. Batista, Marcelo T. de Oliveira, Ataualpa A. C. Braga

**Affiliations:** † Department of Fundamental Chemistry, Institute of Chemistry, University of São Paulo, Av. Prof. Lineu Prestes, 748, São Paulo, São Paulo 05508-000, Brazil; ‡ Institute of Chemistry, 2104 University of Campinas, Monteiro Lobato, 270, Cidade Universitaria,, Campinas, Sao Paulo 13083-862, Brazil; § School of Engineering, Deakin University, Burwood, Melbourne, Victoria 3125, Australia; ∥ School of Life and Environmental Sciences, Deakin University, Burwood, Melbourne, Victoria 3125, Australia; ⊥ Chemistry Institute of São Carlos, University of São Paulo, Av. Trabalhador São Carlense 400, São Carlos, Sao Paulo 13566-590, Brazil

**Keywords:** hydroboration of internal alkynes, computational study, reaction mechanism, regioselectivity, copper, silver, IGM approach

## Abstract

The present study employs DFT calculations and the independent gradient model (IGM) approach to investigate a mechanism study of the hydroboration reaction of internal alkynes catalyzed by Ag­(I)-IMes and Cu­(I)-IMes complexes. A detailed analysis of the mechanism’s steps revealed that Cu­(I)-IMes exhibits superior efficiency, showing a more favorable energy pathway than Ag­(I)-IMes. The IGM method was crucial for quantifying molecular interactions, highlighting essential differences in binding forces between catalysts and substrates throughout the catalytic steps. For Cu­(I)-IMes, the migratory insertion step (TS1) demonstrated a barrier 2.5 times lower than its Ag­(I)-IMes counterpart. Additionally, the protonation step (TS2) exhibited lower energy for Cu­(I)-IMes compared to Ag­(I)-IMes, indicating a more efficient formation of the desired β-product. The results also suggest that Cu­(I)-IMes operates on a more efficient pathway, with lower energy for the catalytic cycle. These findings, coupled with detailed analyses of molecular interactions using the IGM method, provide an enhanced understanding of the reaction mechanism, highlighting the promising efficacy of Cu­(I)-IMes as a catalyst in hydroboration reactions.

## Graphical abstract

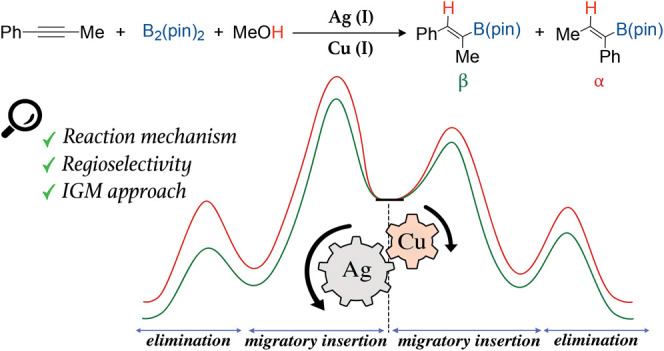

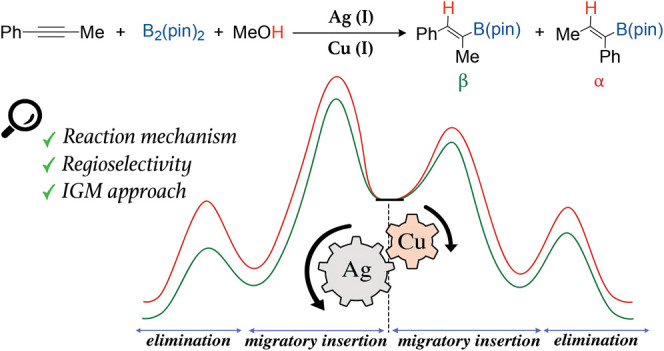

## 1 Introduction

Boron-containing molecules are essential from both academic and industrial standpoints, finding extensive applications in organic synthesis, biologically active agents, and functional molecular systems.^1–4^ Organoboron compounds have consistently demonstrated their role as robust and reliable building blocks, enabling a wide range of transformations in organic and organometallic compounds. The application of transition metals to catalyze the hydroboration reaction has increased in recent years,^5–11^ which has been primarily attributed to incorporating catalysts based on Earth-abundant metals, thereby expanding the scope to encompass a broader range of organic substrates.^12–21^

Over the past decade, significant interest has been drawn toward the catalyzed hydroboration reaction, which serves as a powerful and direct approach to reducing a wide range of unsaturated compounds, including imines, nitriles, carbonyls, alkenes, amides, and even carbon dioxide.^22–25^

More specifically, the selective transformation of alkynes into alkenes through transition metal-catalyzed hydroboration has emerged as a particularly versatile and selective reaction with the application of the resulting alkenyl organoboron compounds in various fields, including organic chemistry, polymers, and agrochemicals.^26,27^

It is possible to find several experimental protocols for transition metal-catalyzed hydroboration^28–33^ and transition metal-free hydroboration^34–37^ in the literature. Among coinage metals, Cu­(I)-catalyzed borylation reactions^38–44^ and Ag­(I)-catalyzed reactions^23,45–49^ have gained substantial attention in recent years.

Yoshida and co-workers pioneered an innovative approach to Ag-catalyzed alkyne hydroboration. They investigated the catalytic hydroboration employing the Ag–NHC (*N*-heterocyclic carbene) complex with alkynes, using B_2_Pin_2_ [bis­(pinacolato)­diboron] and KOtBu in methanol (Scheme 1a). The researchers achieved 85% yield of the hydroborated alkene by using 1-phenyl-1-propyne as substrate and an NHC ligand of the imidazole-2-ylidene type, 1,3-bis­(2,4,6-trimethylphenyl)-1,3-dihydro-2*H*-imidazole-2-ylidene (IMes), to obtain addition at the β-position as the major product. However, when using the ligand 1,3-*bis*(2,6-diisopropylphenyl)­imidazole-2-ylidene (IPr), the reaction did not proceed, indicating its low activity. Interestingly, an impressive yield of 98% was obtained for the same reaction employing a terminal alkyne in the presence of a Cu-IMes catalyst, where, similarly to the Ag catalyst, the β-product was also predominantly obtained.^50^

**Scheme 1:**
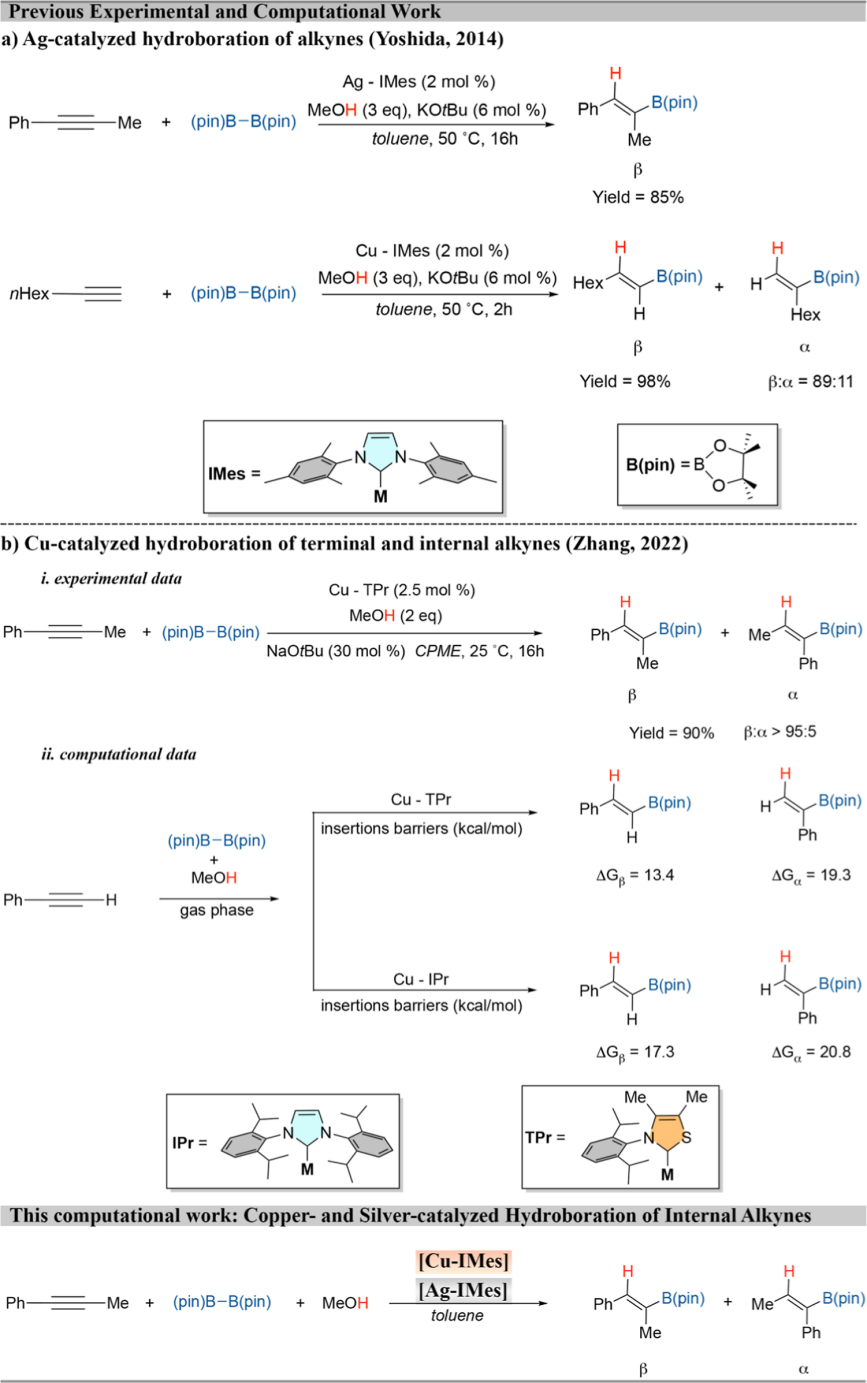
Hydroboration of Alkynes Catalyzed by Ag and Cu Complexes

Recently, Zhang and collaborators reported the first study of an alkyne hydroboration reaction using a Cu–NHC catalyst of the thiazole type, specifically Cu­(I)-thiazol-2-ylidenes (TPr).^51^ They achieved a 90% yield of a trisubstituted vinylboronate through β-hydroboration with greater than 95:5 regio- and Z-selectivity, using 1-phenyl-1-propyne as the substrate. According to the authors, the use of a thiazole ligand results in higher reactivity compared to imidazole-class ligands like IPr. In this context, a computational DFT study was conducted to investigate the hydroboration reaction of a terminal alkyne, addressing the different energies obtained for the same reaction using IPr and TPr ligands. The energy profiles revealed that the IPr ligand requires higher energies in the catalytic cycle, which corroborates with the experimental data indicating its low reactivity (Scheme 1b).^51^

Therefore, the mechanistic understanding of these reactions, especially the factors underlying regioselectivity, is of paramount importance and a crucial step in the further development of such reactions for the borylative transformations of π-systems. While some computational studies on alkyne hydroboration using a monoboron source (H-Bpin) are documented,^32,37,43,52–56^ to the best of our knowledge, mechanistic studies for such reactions employing a diboron source (B_2_pin_2_) remain limited.

Herein, we systematically employed theoretical calculations to investigate the hydroboration reaction of an internal alkyne, catalyzed by Ag­(I)-IMes. Our efforts were dedicated to shed light on the experimental regioselectivity described by Yoshida and colleagues. To expand the scope of our theoretical investigation of the same reaction using an identical substrate, this time employing a Cu-IMes catalyst, we integrated the experimental insights from Yoshida’s research, along with the experimental and theoretical data provided by Zhang et al.^51^ We carried out a comparative effect between Ag and Cu-(IMes) catalysts using precise energies obtained for the catalytic cycle profiles of each catalyst. Furthermore, a qualitative and quantitative analysis of intermolecular interactions based on the independent gradient model (IGM)^57^ method is presented to support the energy data and to investigate the nature of regioselectivity. Thus, this study provides a comprehensive theoretical interpretation of the hydroboration reaction employing B_2_pin_2_, potentially serving as a valuable guide for upcoming related experimental work.

## 2 Theoretical Methods and Computational Details

All density functional calculations were carried out in the Gaussian 09 (Rev. D.01) suite of quantum chemical programs.^58^ We performed geometry optimizations and frequency calculations in the solvent phase using the hybrid density functional B3LYP^59–61^ with semiempirical D3 ^62,63^ dispersion corrections (B3LYP-D3), incorporating solvent effects via the continuum solvation model SMD^64^ for toluene. The SDD (Stuttgart/Dresden) quasi-relativistic pseudopotential^65^ and associated basis set were applied to the metal atoms (Ag or Cu), while the 6-31G­(d,p)^66^ basis set described all other atoms. Vibrational frequency calculations were performed to determine that the local minima have zero imaginary frequencies and the transition state (TS) structures have exactly one imaginary frequency corresponding to the desired eigenmode. TS structures were also verified by intrinsic reaction coordinate (IRC) analysis.^67,68^ To improve accuracy, energies were also computed by applying the domain-based local pair natural orbital coupled-cluster theory, including singles, doubles and “semi-canonical” perturbative triples approximation, known as DLPNO–CCSD­(T0),^69,70^ together with the def2-TZVP^71^ atomic basis set and matching auxiliary basis sets. Predefined thresholds, including NormalPNO, were requested in the ORCA 4.2 program.^72^ Free energies reported in the text refer to electronic energies obtained with DLPNO–CCSD­(T0)/def2-TZVP, which were corrected by free energy contributions at 298.15 K and solvent contributions at 1 mol·L^–1^ obtained with DFT computations [Δ*G*_298.15K, SMD, DLPNO–CCSD(T0)_].

The recently introduced topological analysis based on the electron density ρ (ED) descriptor δg^inter^ interaction score^73,74^ and intrinsic bond strength index (IBSI) index^75^ were used in the corresponding transition state geometries to identify and quantify molecular interactions. Similarly to the noncovalent interaction analysis (NCI) approach, the independent gradient model (IGM) approach^57^ provides an intuitive spatial map of local repulsive, nonbonding, and attractive interactions materialized by isosurface density gradients. However, unlike NCI, the IGM approach can quantify the interaction between two fragments through descriptor, δg^inter^.^76^ Additionally, it provides a score that internally probes the strength of a given pair of atoms in a molecular situation, IGM-δg^pair^, through the IBSI. The resulting δg isosurfaces, representing the interaction regions, are colored according to the ED value using the sign of the second eigenvalue of the ED Hessian (λ_2_). A blue–green–red color code is then used, as follows: blue for strongly attractive, green for van der Waals, and red for strongly repulsive interactions.

We also investigated the origin of regioselectivity in the Cu- and Ag-catalyzed systems. For this and also to support the energy data, we performed bond strength analysis based on the IBSI obtained by IGM approach. The IGMPlot code^57^ with quantum mechanical electron density (B3LYP-D3/def2-TZVP) was applied for bond strength analysis based on the IBSI by IGM approach. The qg = |∇ρ^IGM^|/|∇ρ| descriptor, with ∇ρ^IGM^ being the upper limit of the ∇ρ for the ED gradient, is used in the IGMplot to color points in the δg­(ρ) plots. The 3D isosurface representations were generated using the VMD software.^77^ Molecular structures were prepared using CYLView (http://www.cylview.org).^78^

A brief general description of the computational methods is included in the Supporting Information, as suggested by one of the reviewers.

## 3 Results and Discussion

### 3.1 Catalytic Cycle by Copper- and Silver-Complex, as Proposed by Yoshida

Inspired by Yoshida’s experimental investigations,^50^ we have proposed a plausible mechanism for hydroboration of alkynes catalyzed by Ag. We extend the same mechanistic framework to the analogous reaction catalyzed by Cu. As illustrated in Scheme 2, the initial step (**I**) involves insertion of the alkyne into the catalytic active species (**Ag**-**CAT** and **Cu-CAT**) through transition state **TS1**, affording intermediate (**Int2**). This intermediate is subsequently protonated via the addition of MeOH at **TS2**, yielding hydroboration product. The protonation of **Int2** then yields the hydroboration products through an elimination process (**II**). In these two primary stages, two distinct pathways are conceivable, dictating the regioselectivity of the reaction, either positioning the phenyl group on the same carbon as the B­(pin) moiety, denoted as the α-position, or on the adjacent carbon, designated as the β-position. The concluding step (**III**) encompasses the σ-bond metathesis between the metal alkoxide and diboron, B_2_(pin)_2_, regenerating the active catalytic species and yielding MeO–B­(pin) as a reaction byproduct (**TS3**).

**Scheme 2:**
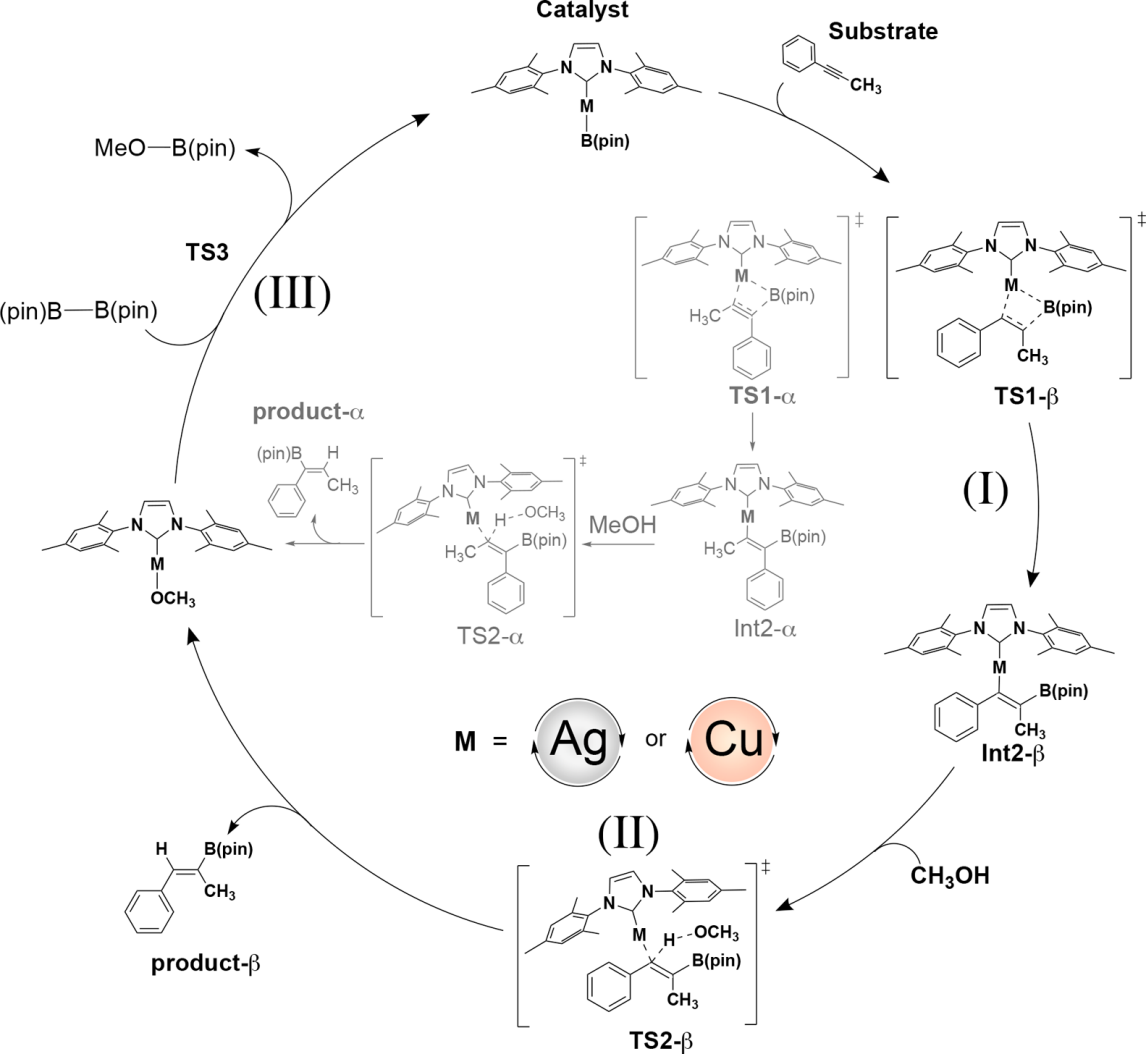
Plausible Catalytic Cycle of Copper- and Silver-Catalyzed Hydroboration of Alkynes^a^

a (**I**) insertion of the alkyne into the catalytic active species (**Ag**-**CAT** and **Cu-CAT**) through transition state **TS1**, affording intermediate (**Int2**). (**II**) protonation via the addition of MeOH at **TS2**, yielding hydroboration product. (**III**) σ-bond metathesis between the metal alkoxide and diboron, B_2_(pin)_2_, regenerating the active catalytic species and yielding MeO–B­(pin) as a reaction byproduct (**TS3**)

Despite the previous experimental exploration of Ag and Cu-catalyzed hydroboration reactions with internal alkynes, no computational investigation has been reported. Thus, we calculated the catalytic cycles using DFT to elucidate the mechanisms and understand the origin of the selectivity. The catalytic cycles of both Ag and Cu were modeled based on Yoshida’s proposal.^50^ Thus, we considered elaborate mechanisms within the closed-shell singlet state for Ag (I) and Cu (I), with the catalytic cycle originating from the active **CAT**.

We performed a study to discern the relative efficacy of Ag and Cu catalysts in internal alkyne hydroboration. This investigation sought to determine which catalyst demonstrates superior performance. Toward this end, we leveraged energy data obtained through quantum calculations and supplemented our analysis with quantitative insights derived from the IGM approach, which characterizes the strength of molecular interaction.

### 3.2 Silver-Catalyzed Hydroboration of Internal Alkynes

#### 3.2.1 Reaction Mechanism

As shown in Figure 1, the Ag­(I) catalyst (**Ag-CAT**) and the substrate, 1-phenyl-1-propyne (**1**), gradually attracted each other to form the Ag-Int1 adduct. However, this first step produces two possible complexes as intermediates. The first possibility is the insertion at the α-position, denoted as **Ag-Int1-α**. The alkyne triple bond at the α-position located near the Ag­(I) center is activated, and the C–B and C–Ag bonds facilitate a concerted migratory insertion through the transition state **Ag-TS1-α**, associated with a barrier of 26.5 kcal/mol and resulting in the formation of the **Ag-Int2-α** intermediate. The second possibility refers to the β-position insertion (**Ag-Int1-β**). The computational results indicate that **Ag-TS1-β** displays lower energy levels with a barrier of 21.1 and Δ*G* ^‡^ = 28.2 kcal/mol. Consequently, **Ag-TS1-β** is 6.3 kcal/mol more stable than its α-position counterpart (Figure 2). Subsequently, the protonation step takes place through the addition of methanol across the borylalkenyl M–C bond. As evidenced by the energy profile in Figure 1, protonation of **TS2-β** is more favorable than that of **Ag-TS2-α** (Figure 2). The Gibbs free energy of the favored **Ag-TS2-β** leads to the experimentally observed major hydroboration product (product-β).^50^ In the favored transition states (**Ag-TS1-β** and **Ag-TS2-β**), the phenyl group positions away from the B­(pin), on the adjacent carbon, while in the disfavored TSs, it is on the same carbon bearing the bulky B­(pin) ligand. The calculations suggest that higher energies are due to steric hindrance. Moreover, the elimination step dictates the stereoselectivity. These results corroborate the experimental observation, which predominantly yields the β-product.

**Figure 1:**
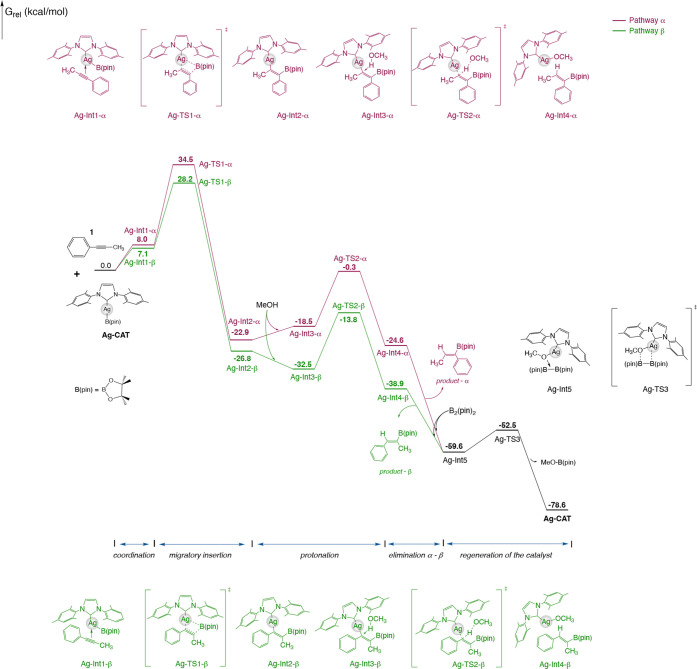
Gibbs free energy diagram (in kcal/mol) for the silver-catalyzed hydroboration of the alkyne 1. Pathway α is shown in red, while pathway β is shown in green.

**Figure 2:**
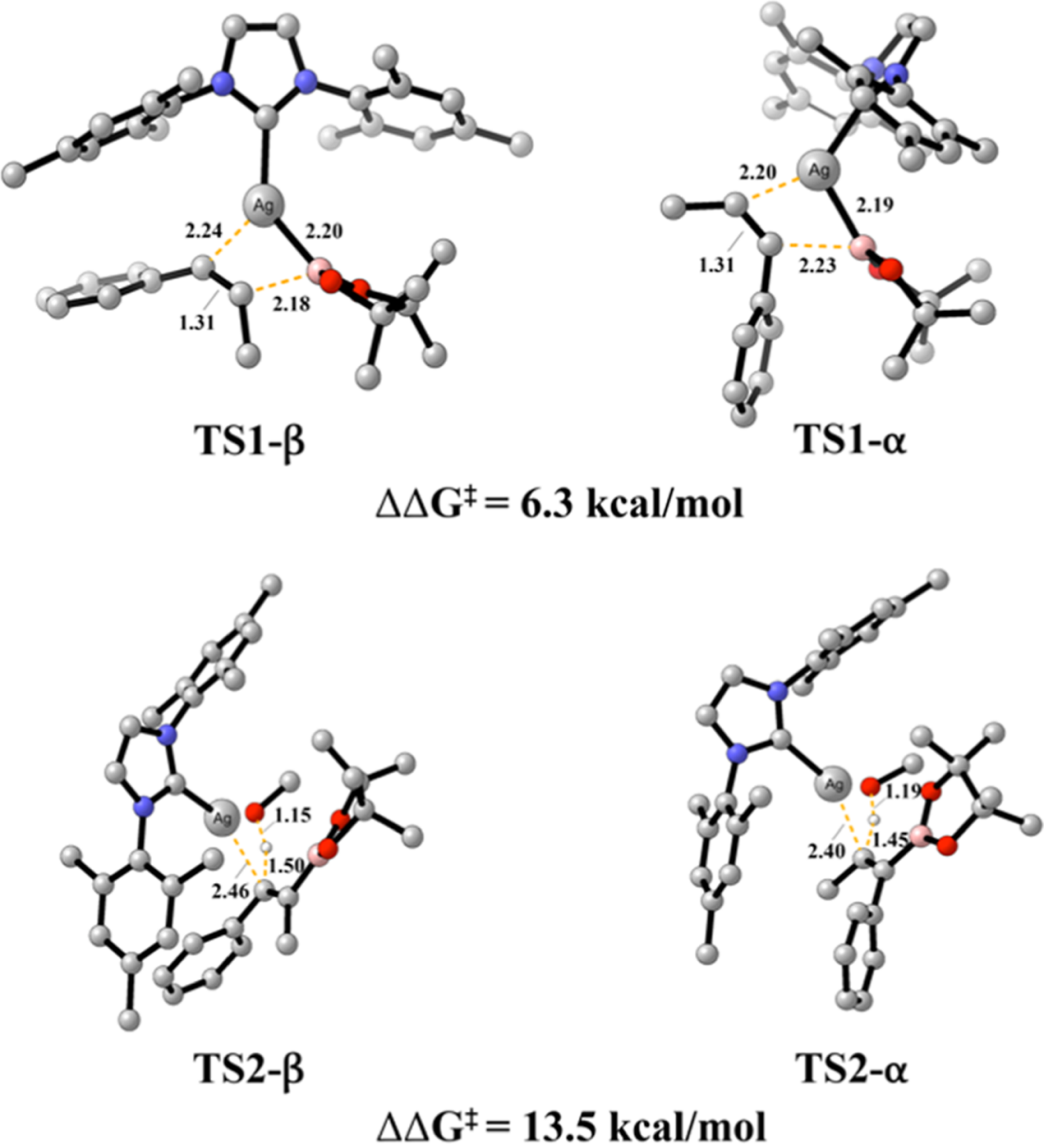
Relative Gibbs free energies for α and β TSs in the migratory insertion and protonation steps of the Ag-catalyzed mechanism.

Finally, catalyst regeneration occurs through a four-membered transition state, **Ag**-**TS3**, with an activation free energy of 7.1 kcal/mol. The mechanism proceeds in a concerted regime, yielding MeO–B­(pin) as a byproduct and effectively regenerating the active catalytic species, **Ag-CAT**.

#### 3.2.2 Origins of Regioselectivity

The initial two steps of the reaction mechanism are directly involved with the α/β-regioselectivity (Figure 3). For **TS1**, it is noteworthy that although the distance between Ag and B atoms varies by only 0.01 Å between the α- and β-positions, the bond strength between these atoms favors **TS1-α** (IBSI = 0.229) over **TS1-β** (IBSI = 0.221). This distinction is also reflected in the subtle change in the isosurfaces, with **TS1-α** predominantly appearing in blue. As for the C–B bond, the shorter bond distance in **TS1-β** (2.18 Å) results in a higher bond strength (IBSI = **0.191**) compared to TS1-α (IBSI = **0.176** exhibits indices following the increased stabilization of the transition state with the breaking of the Ag–B bond and the formation of the C_2_–B bond. In the protonation step, the interactions involving the breaking of the Ag–C_1_. The stronger bond between Ag–O, with IBSI = 0.123 and the lower IBSI index for the Ag–C_1_ bond (IBSI = 0.129 exhibits a stronger bond (characterized by more attractive interactions) between Ag and C_1_ atoms (IBSI = **0.150**), rendering the cleavage of this bond more challenging than the β-regioisomer.

**Figure 3:**
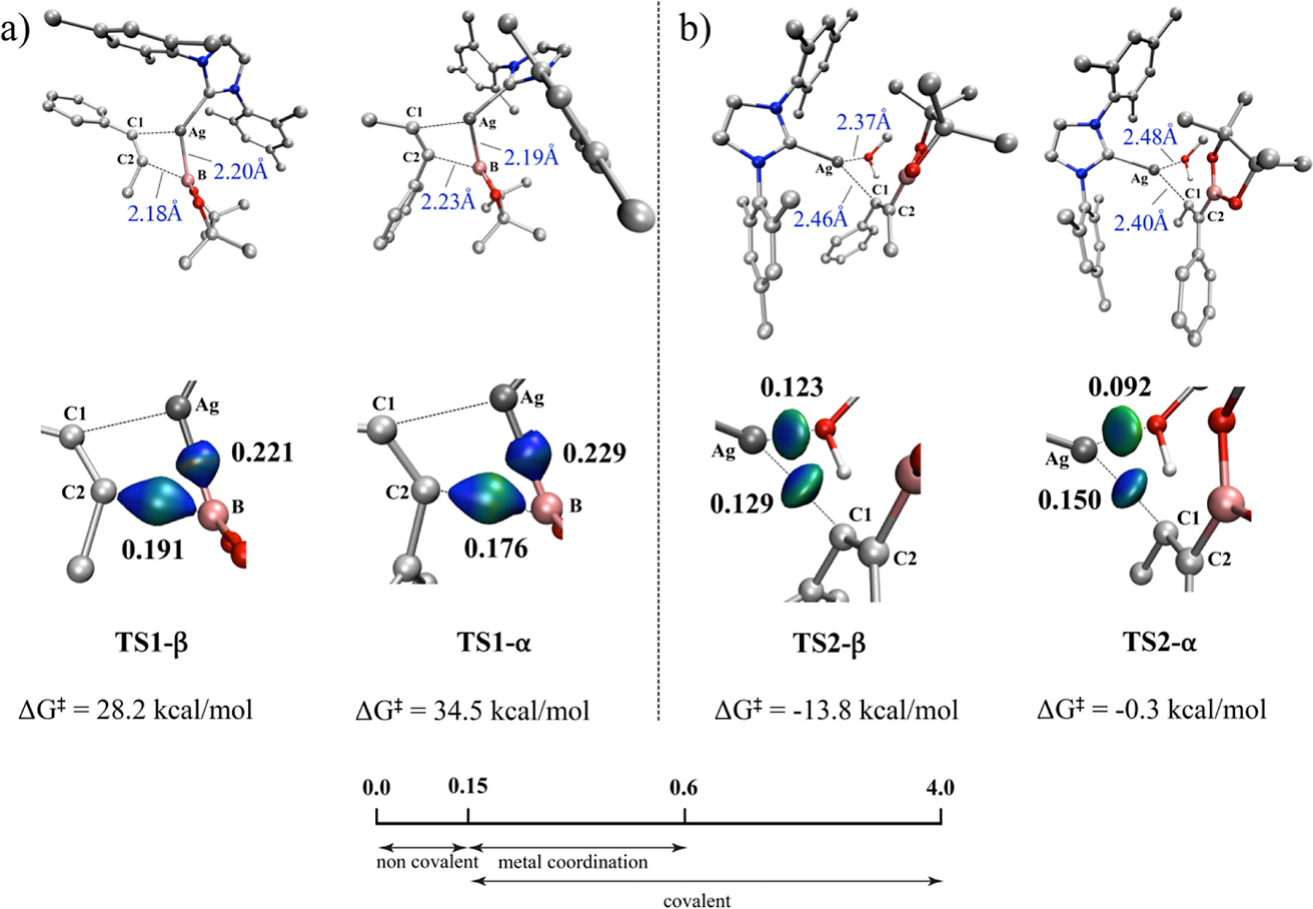
Bond distances and associated bond strength index (IBSI, bold) for α and β (**a**) **TS1** and (**b**) **TS2**. δg^pair^ = 0.045 a.u isosurfaces for selected bonds in TSs involving a silver complex (B3LYP-D3/def2TZVP); color coding in the range −0.08 < sign­(λ_2_)­ρ < 0.08 au Indicative IBSI scale and isosurface color-coding, as follows: blue for attractive interactions, green for weak interactions and red for repulsive interactions.

These findings are aligned with the bond distances and energies determined for each transition state.

### 3.3 Copper-Catalyzed Hydroboration of Internal Alkynes

#### 3.3.1 Reaction Mechanism

The first step involves the migratory insertion of the active specie (**Cu-CAT**) into the alkyne triple bond, 1-phenyl-1-propyne (**1**), leading to the formation of the **Cu-Int2** adduct (Figure 4). The barrier for this step is 11.2 kcal/mol for **Cu-TS1-α** and 8.3 kcal/mol for **Cu-TS1-β**. Consequently, **Cu-TS1-β** presents a distinct stability advantage of ΔΔ*G* ^‡^ = 3.9 kcal/mol (Figure 5).

**Figure 4:**
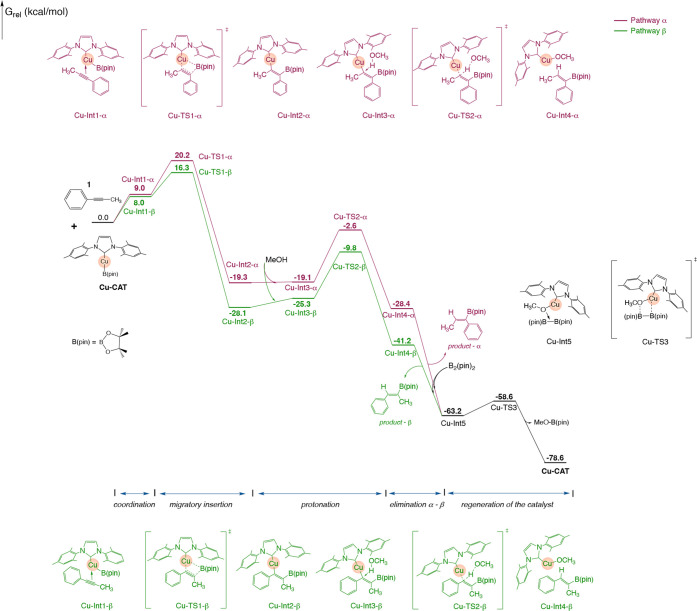
Gibbs free energy diagram (in kcal/mol) for the copper-catalyzed hydroboration of the alkyne 1. Pathway α is shown in red, while pathway β is shown in green.

**Figure 5:**
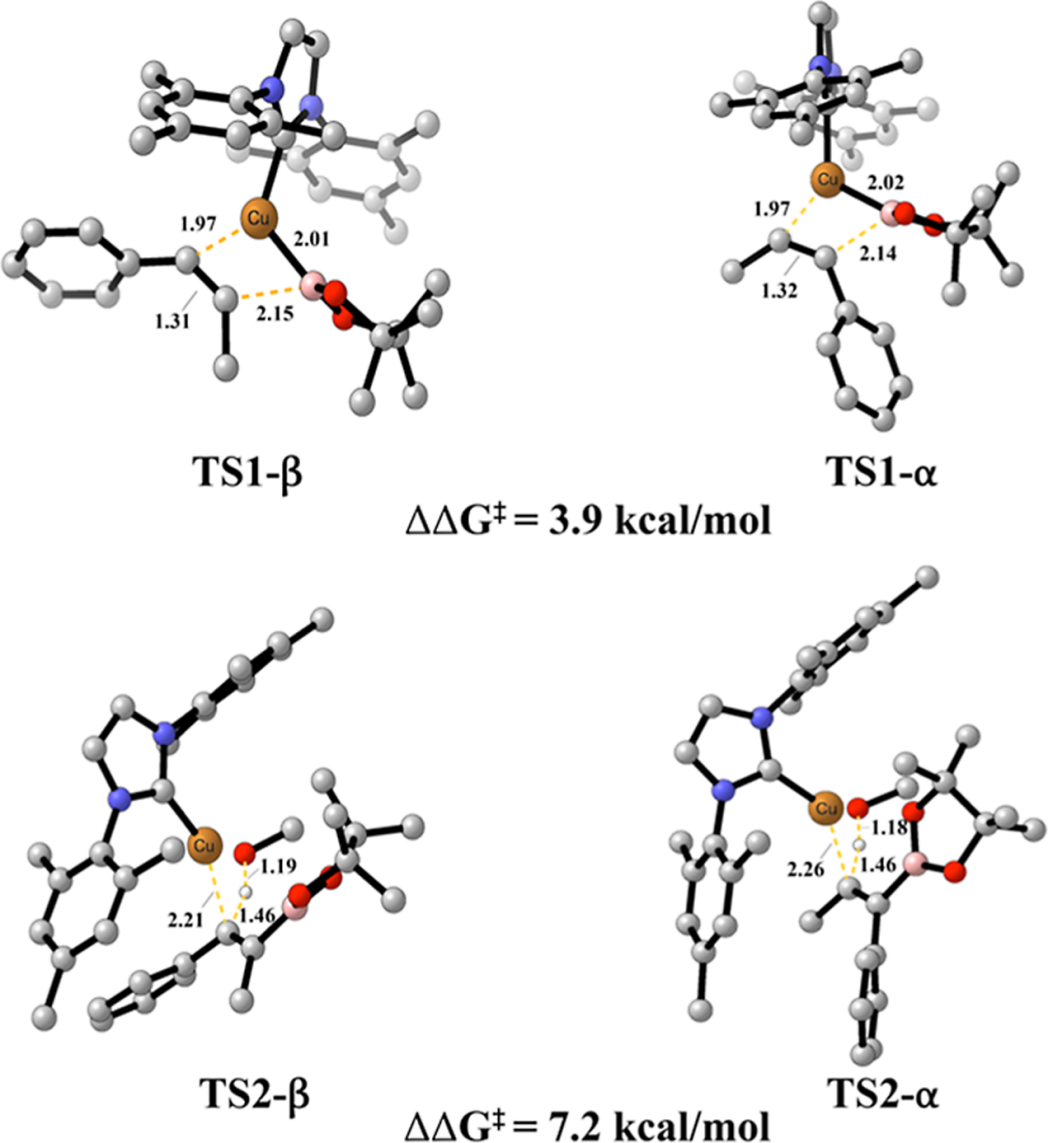
Relative Gibbs free energies for α and β TSs in the migratory insertion and protonation steps of the Cu-catalyzed mechanism.

The concept distinguishing between the α- and β-positions remains consistent with the explanation provided in Section 3.2. The protonation step occurs through methanol addition via transition state **TS2**, associated with a barrier of 16.5 kcal/mol for α-protonation and 15.5 for β-protonation, Figure 4. The thermodynamic energies shows that protonation via **Cu-TS2-β** is more favorable than protonation via **Cu-TS2-α**, ΔΔ*G* ^‡^ = 7.2 kcal/mol (Figure 5).

The calculations with copper indicate that higher energies result from the same steric hindrance observed for Ag analogs and that the elimination step dictates regioselectivity, mainly producing the β-product. These findings corroborate earlier computational studies on the hydroboration of terminal and internal alkynes, which showed the impact of steric effects on the regioselectivity of hydroboration reactions favoring β-product formation.^51^ The tendency is also observed for catalysts containing other metals.^25^

Next, catalyst regeneration takes place through addition of B_2_(pin)_2_, generating a four-membered transition state, **Cu-TS3** with an activation free energy of 4.6 kcal/mol. This process operates in a concerted manner, in which formation and breaking of bonds occur simultaneously, yielding the MeO–B­(pin) species as a byproduct and restoring the active catalytic species, **Cu-CAT**. Additionally, the results are compelling evidence that the catalytic cycle for the hydroboration reaction of internal alkynes is exergonic, with an overall Δ*G* of −78.6 kcal/mol. The elimination step governs stereoselectivity.^25^

#### 3.3.2 Origins of Regioselectivity

As depicted in Figure 6a, the bond distance between C1 and Cu atoms is consistent at 1.97 Å for both transition states. However, the interaction in **TS1-β** is slightly stronger (IBSI = 0.367), resulting in lower energy than **TS2-α** by 3.9 kcal/mol.

**Figure 6:**
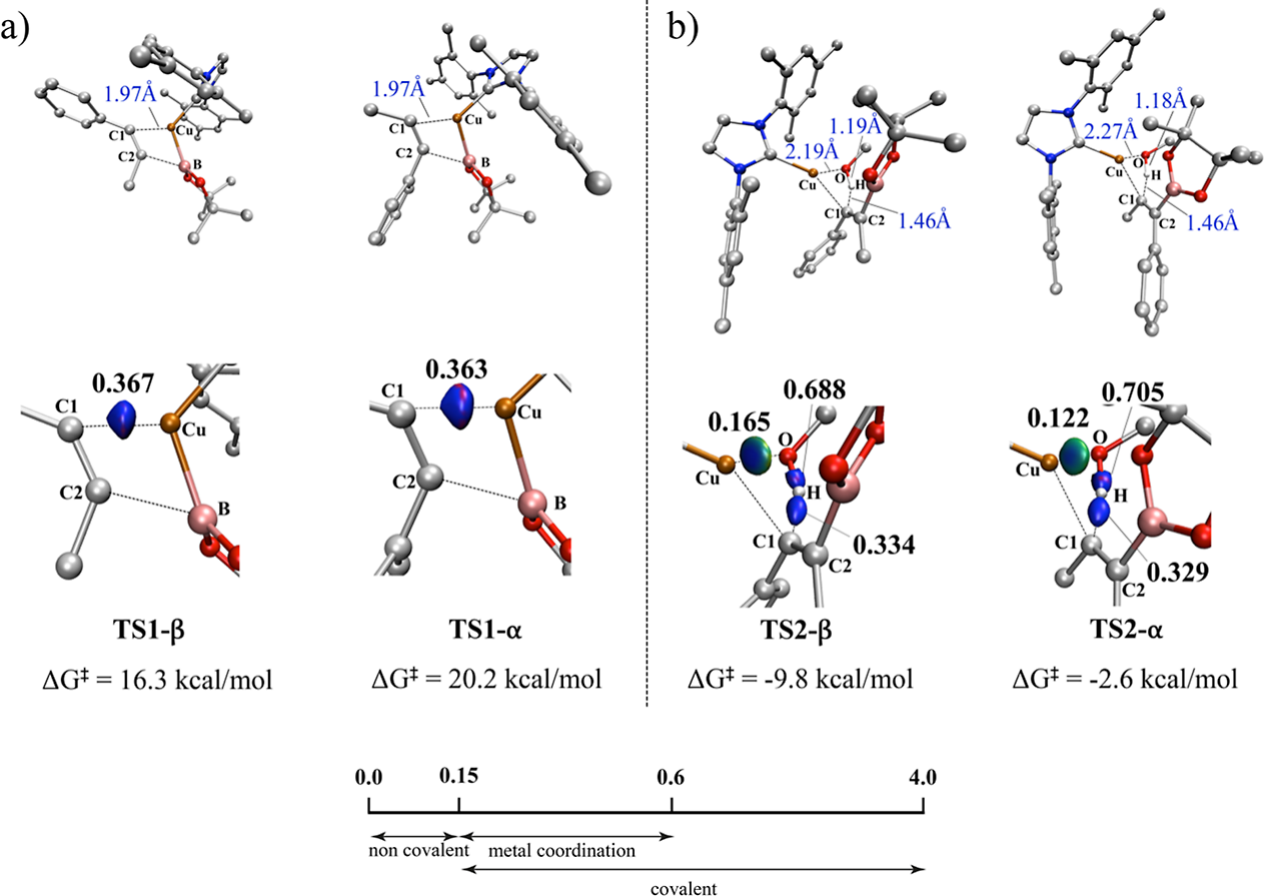
Bond distances and associated bond strength index (IBSI, bold) for α and β (a) **TS1** and (b) **TS2**. δg^pair^ = 0.045 a.u isosurfaces for selected bonds in TSs involving a copper complex (B3LYP-D3/def2TZVP); color coding in the range −0.08 < sign­(λ_2_)­ρ < 0.08 au Indicative IBSI scale and isosurface color-coding, as follows: blue for attractive interactions, green for weak interactions and red for repulsive interactions.

For the protonation step (Figure 6b), we observe that the bond strength between H and C1 atoms is higher in **TS2-β** (0.334 versus 0.329). This observation aligns with the energy data obtained from our quantum calculations, indicating that the β-position of the alkene is more accessible. Consistently, the bond between O and H atoms in methanol exhibits a lower covalent character for **TS2-β** (IBSI = 0.688), requiring less energy to break compared to the α-position (IBSI = 0.705). As a result, we observe a stronger interaction between Cu and O atoms in **TS2-β** (IBSI = 0.165), in an energetically more favorable pathway than its α-position counterpart.

### 3.4 Cu-(IMes) and Ag-(IMes): IGM Approach and Energy Data

After providing a comprehensive description of the mechanisms and effects of regioselectivity in the hydroboration reaction of 1-phenyl-1-propyne catalyzed by Ag- and Cu- (IMes), we present a recent approach that highlights the main differences between the catalysts studied in this work. Here, we will consider only the β-position geometries, which exhibit pathways of lower energy, as explained in Sections **2.2.1** and **3.3.1**. Figure 7 shows a comparative overview of the energy profiles for the reaction under investigation. We can observe that, while the Ag-catalyzed reaction follows a plausible pathway, it is noteworthy that the reaction path for Cu catalysis follows a lower energy route.

**Figure 7:**
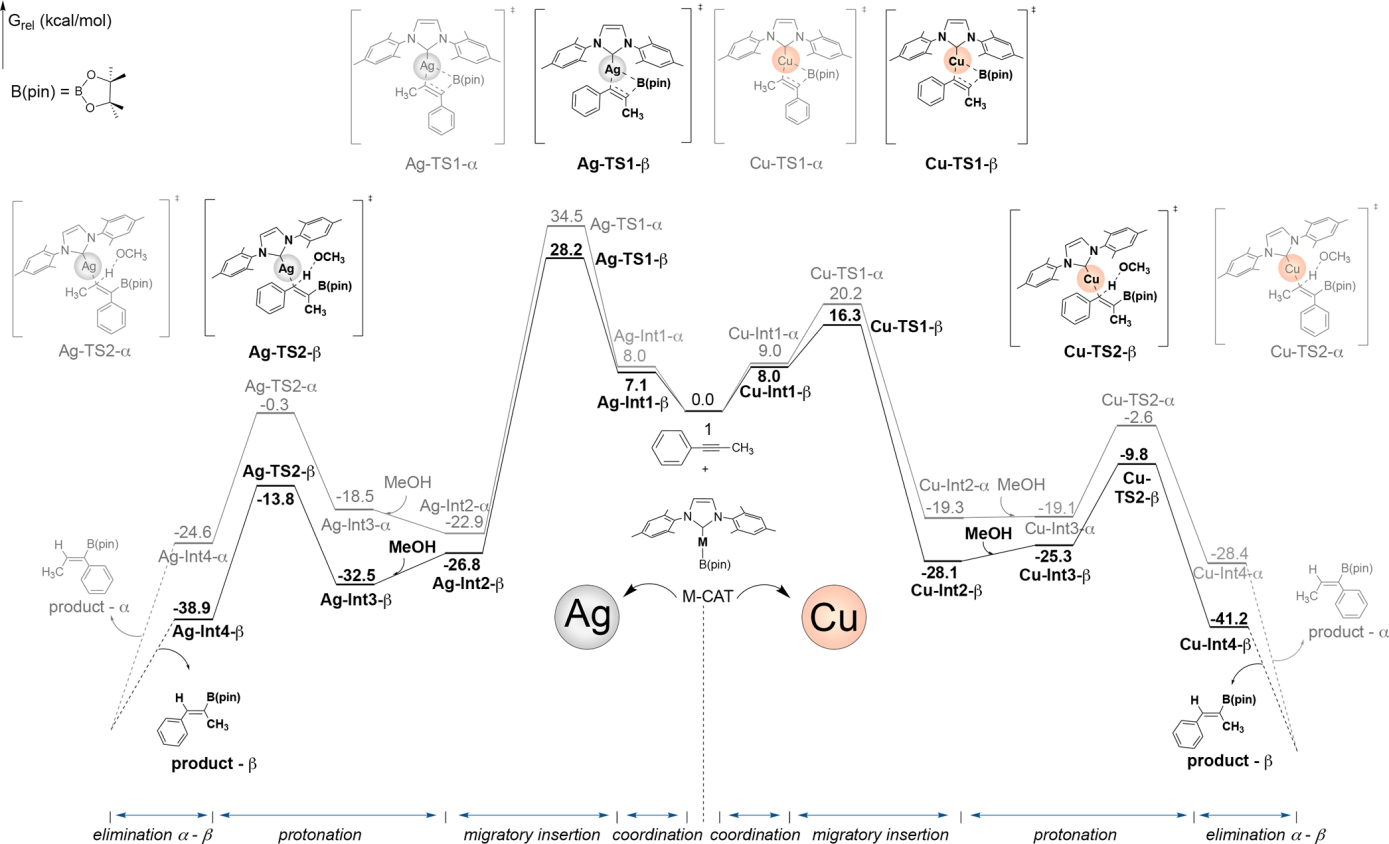
Comparative Gibbs free energy profile for the hydroboration of 1-phenyl-1-propyne catalyzed by Ag- and Cu-complexes (first two steps shown).

For the Ag (IMes)-catalyzed reaction, the highest energy barrier is 21.1 kcal/mol and occurs in the migratory insertion step between Ag-TS1 and Ag-Int1. In contrast, for the Cu-catalyzed reaction, the barrier associated with the same step is only 8.3 kcal/mol, 2.5 times lower than its Ag analog. In the protonation step, even though the energy of Cu-TS2 is slightly higher than Ag-TS2, the barrier associated with this step favors the elimination of the alkene with IMes-Cu by 3.2 kcal/mol.

Thus, the mechanism for the 1-phenyl-1-propyne reaction in the presence of Cu-IMes catalyst described here follows a more favorable energy pathway than the Ag-IMes catalyst. Despite using a different substrate, the energy obtained for the protonation step followed by β-elimination is similar to the barriers associated with using a highly efficient Cu-(TPr) catalyst.^51^

In order to clarify the role of intermolecular interactions involved in TS1, we performed an analysis of noncovalent interactions by using the local IGM-δg^inter^ descriptor. The molecular fragments, as well as the strength of the intermolecular interactions are described in Table 1.

**Table 1:**
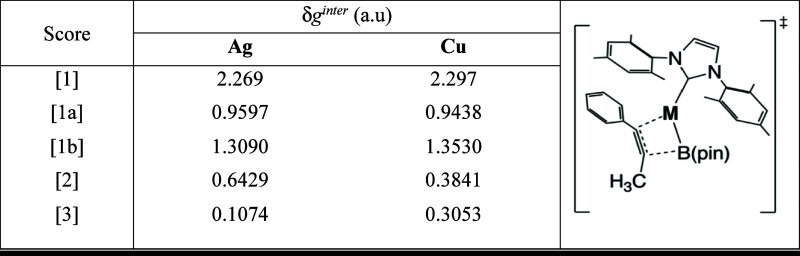
Inter-fragment Analysis.^a^

a FRAG1:1-phenyl-1-propyne.

b FRAG2: catalyst with **M** = **Ag** or **Cu**.

c IGM scores for **TS1** using QM treatment. **[1]** represents full [1a] + [1b]; **[1a]** non-bonding interaction (λ_2_ > 0); **[1b]** bonding interaction (λ_2_ < 0); **[2]** weak interactions; **[3]** strong interactions (covalent).

After establishing the effects governing selectivity, we focus on the β-position to analyze the interactions involving silver and copper catalysts. Therefore, we chose to assess the interactions involved in the highest energy transition state (**TS1**), which includes the alkynyl moiety and the catalyst.

The δg^inter^ [1] score corresponds to the addition of all interactions between fragments (0 < λ_2_ < 0). Accordingly, we observe that the Cu-catalyst exhibits a lower repulsive interaction ([1a] = 0.9438) and a stronger attractive interaction ([1b] = 1.3530) compared to the Ag-catalyst, resulting in a higher δg^inter^ [1] (2.297).

Focusing on attractive interactions (λ_2_ < 0), we observe that the interaction between the alkynyl group and the active catalytic species has a more pronounced covalent character for the Cu-(IMes) ([3] = 0.3053), which is nearly three times greater than the same interaction for the Ag-(IMes) ([3] = 0.1074). This result corroborates the greater stabilization of **TS1-Cu**, which exhibits an energy value that is more than 10 kcal/mol lower than that of **TS1-Ag** (16.3 versus 28.2 kcal/mol).

It is worth noting that the scores quantifying only attractive interactions (λ_2_ < 0) are extracted from the 2D-plot signature, (Figures 8a and 9a). It is clear that Cu-IMes exhibits stronger interactions, particularly between the metal and C1 atoms when compared to the same Ag-catalyst. The isosurfaces reflect the values, showing the highest density for the Cu–C1 interaction (Figures 8b and 9b).

**Figure 8:**
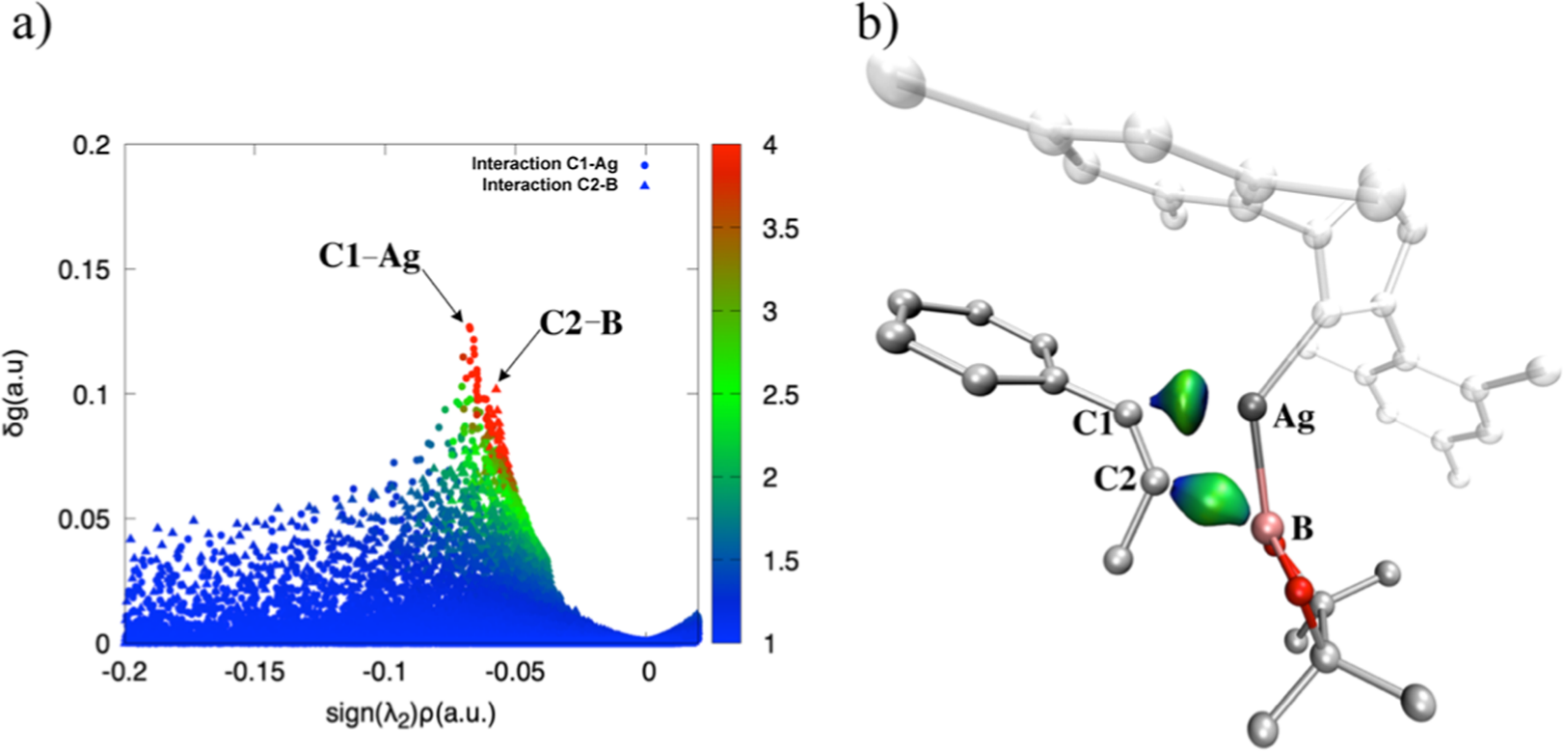
(**a**) IGM-δg^inter^ 2D-plot colored according to qg descriptor in the range: 1 < qg < 4. (**b**) IGM-δg^inter^ = 0.03 au isosurfaces obtained for the Ag – C1 and C2 – B fragments with BGR color code in the range −0.2 < sign­(λ_2_)­ρ < 0.2 au **Ag-TS1-β** obtained by B3LYP-D3/def2-TZVP level of theory.

**Figure 9:**
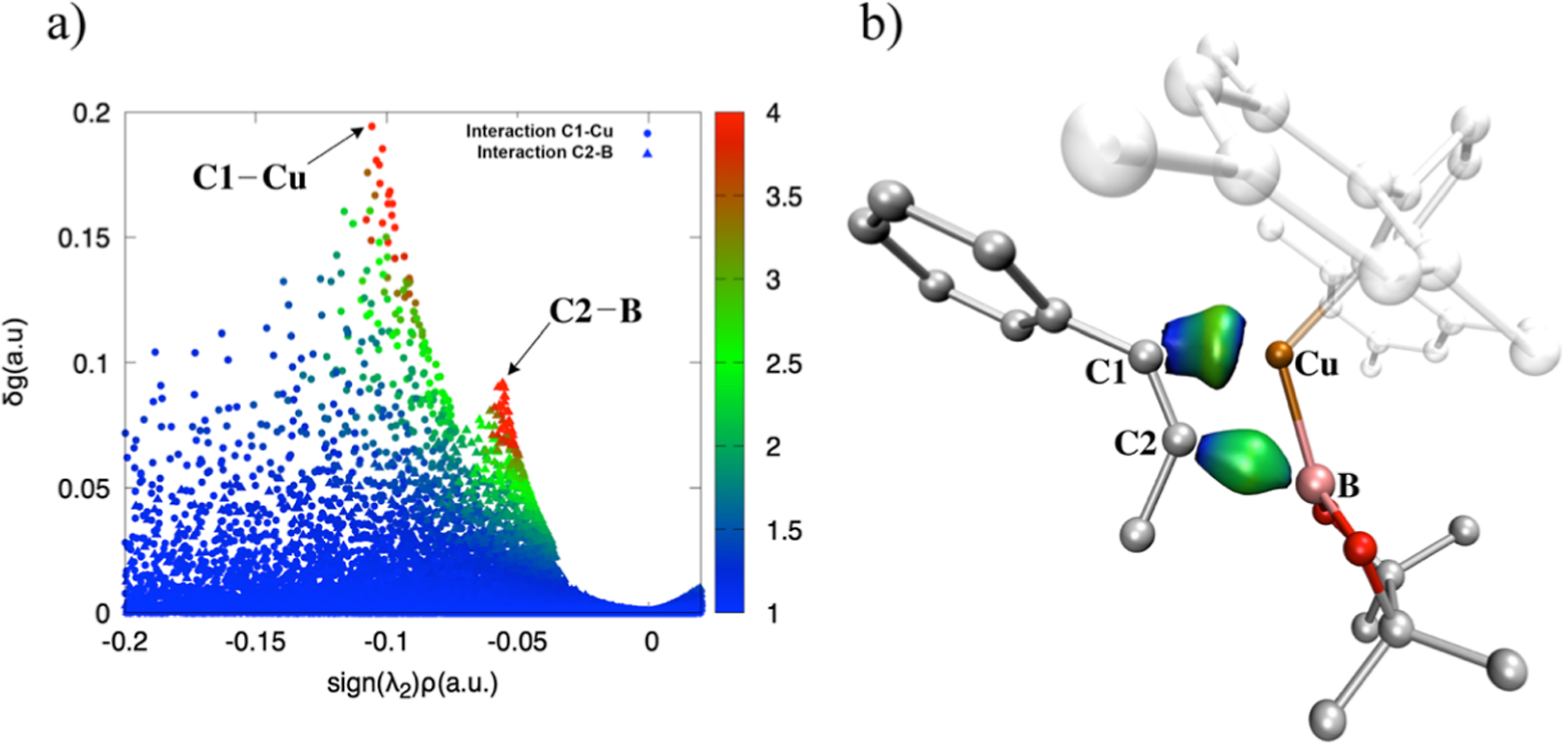
(**a**) IGM-δg^inter^ 2D-plot colored according to qg descriptor in the range: 1 < qg < 4. (**b**) IGM-δg^inter^ = 0.03 au isosurfaces obtained for the Cu – C1 and C2 – B fragments with BGR color code in the range −0.2 < sign­(λ_2_)­ρ < 0.2 au **Cu-TS1-β** obtained by B3LYP-D3/def2-TZVP level of theory.

Therefore, the results based on DFT calculations and interaction analyses using the IGM-approach indicate that the Cu-catalyst demonstrates higher catalytic efficiency compared to the Ag-catalyst for the hydroboration reaction of 1-phenyl-1-propyne in the presence of methanol and B_2_(pin)_2_. The π-backdonation effect metal–ligand is closely associated with the observations made here, leading us to conclude that electronic effects are responsible for the energy differences obtained for the Ag and Cu catalysts, as shown by Nolan et al.^79^ In their study, the authors described the M-C_Carbene_ bond in M-NHC complexes as a coordination bond formed by the interaction between the lone pair of electrons on the carbon atom in the heterocyclic carbene and the empty orbital of the metal. Although the lone pair on the carbon atom of the NHC acts as a strong σ-donor, enabling the formation of stable metal-NHC complexes with most transition metals, the weak π-backdonation ability of silver, compared to other transition metals, results in a weaker Ag–C_Carbene_ bond than typically observed in M-C_Carbene_ bonds of other metal-NHC complexes, such as those involving Cu. This difference is partly attributed to bond length, as the Ag–C_Carbene_ bond is longer than M-C_Carbene_ bonds in comparable metal-NHC complexes, thereby reducing the Lewis acidity of Ag­(I).^79^

The insights presented herein have the potential to furnish valuable guidance to experimental research groups employing transition metal catalysts in exploration boron incorporation reactions within unsaturated substrates.

## 4 Conclusions

In summary, this study has provided a detailed theoretical investigation into the hydroboration reaction of internal alkynes, catalyzed by both Ag­(I)-IMes and Cu­(I)-IMes complexes. Through DFT calculations, we have clarified the reaction mechanisms, origins of regioselectivity, and the comparative effectiveness of Ag and Cu catalysts. The hydroboration process proceeds through a multistep catalytic cycle, in which the initial insertion of the alkyne into the M–B bond and subsequent protonation and elimination steps are crucial in determining the reaction’s regioselectivity.

Both Ag and Cu catalysts favor the formation of β-products due to lower energy barriers and more stable intermediates at the β-position, corroborated by experimental data. The regioselectivity is primarily governed by steric effects, as the α-position experiences higher energy barriers during the insertion and elimination steps. IGM analysis through the IBSI index supports this finding. Additionally, Cu-IMes catalysts exhibit lower energy pathways compared to Ag-IMes catalysts, making them more efficient. The energy barrier for the migratory insertion step is significantly lower for Cu (8.3 kcal/mol) compared to Ag (21.1 kcal/mol), further supporting the superior catalytic performance of Cu. The weaker Lewis acidity of Ag reduces its interaction strength with the substrate and limits its ability to stabilize key intermediates, explaining the difference in efficiency.

Furthermore, noncovalent interaction analysis reveals that Cu-catalysts have stronger covalent character interactions and less repulsive forces than Ag-catalysts, accounting for the greater stabilization and lower energy profiles of the Cu-catalyzed reactions. Additionally, our findings align with previous studies on copper-catalyzed borylation, which highlight the important roles of steric bulk and Lewis acidity in determining regioselectivity.^80,81^

Ultimately, results herein provide crucial mechanistic insights and quantitative data, which can serve as a guide for future experimental efforts in the field of alkyne hydroboration, particularly with regard to the optimization of catalytic processes. Furthermore, the IGM study sheds light on the strength of M–L interactions, thereby explaining the higher efficiency of Cu catalysts over Ag.

## Supplementary Material



## Data Availability

The data underlying this study are available in the published article and its Supporting Information.

## Notes

Tables containing computed energies and geometries for all reaction intermediates and transition states (PDF)
