# Supplementary material for: Mechanistic Study on Copper- and Silver-Catalyzed Hydroboration of Internal Alkynes: A DFT Study
Source: ACS Org Inorg Au. 2025 Mar 11;5(3):181–93. doi: 10.1021/acsorginorgau.5c00004 (PMC12142438; doi:10.1021/acsorginorgau.5c00004)
Supplement: Supplementary file 1 [file gg5c00004_si_001.pdf]

Supporting Information

Mechanistic Study on Copper- and Silver-catalyzed Hydroboration of Internal Alkynes: A DFT Study

Ivanna G. R. Juliani Costa,[1] Patrick R. Batista,[2] Marcelo T. de Oliveira,[3,4,5] Atauapa A. C. Braga. *[1]

[1]*Department of Fundamental Chemistry, Institute of Chemistry, University of Sao Paulo, Av. Prof. Lineu Prestes, 748, Sao Paulo, SP 05508-000, Brazil.*
[2]*Institute of Chemistry, University of Campinas, Monteiro Lobato, 270, Cidade Universitaria, 13083-862, Campinas, Sao Paulo, Brazil.*
[3] *School of Engineering, Deakin University, Burwood, Melbourne, Victoria 3125, Australia.*
[4] *School of Life and Environmental Sciences, Deakin University, Burwood, Melbourne, Victoria 3125, Australia.*
[5] *Chemistry Institute of São Carlos, University of São Paulo, Av. Trabalhador São Carlense 400, 13566-590, São Carlos, SP, Brazil*

E-mail: ataualpa@iq.usp.br

General Summary of the Computational Methods Used: In this study, we utilized well-established computational methods based on density functional theory (DFT) to investigate the reaction mechanisms of copper- and silver-catalyzed hydroboration of alkynes. Additionally, we employed high-level quantum mechanical methods based on coupled-cluster theory to refine the energy calculations. Solvent effects were included to better approximate real experimental conditions. We also analyzed molecular interactions using specialized techniques (NCI and IGM) that visualize attractive and repulsive forces within the system. This helped us understand how different factors influence regioselectivity—the preference for forming one product over another.

Content

I. COMPUTED ENERGIES S1
II. CARTESIAN COORDINATES S5

I. Computed Energies

Table S1: Energies by optimization calculations. Electronic Energy (EE); Sum of electronic and zero-point Energies (SZPE); Sum of electronic and thermal Enthalpies (SE) and Sum of electronic and thermal Free Energies (SFE) for Ag-system.

	EE[a] (a.u)	SZPE[a] (a.u)	SE[a] (a.u)	SFE[a] (a.u)	EE $_{gas-phase}$[b] (a.u)
**substrate**	-347.739577425	-347.601284	-347.591969	-347.636826	-347.728404285
**B_2(pin)$_2$**	-822.638119861	-822.273177	-822.252883	-822.319758	-822.625239762
**MeOH**	-115.727798327	-115.676557	-115.672291	-115.699308	-115.725088573
**product**	-759.739850806	-759.404662	-759.385912	-759.450297	-759.694389229
**MetOBpin**	-526.506194703	-526.280817	-526.267629	-526.318100	-526.498365823
**Ag-Catalyst**	-1482.64478511	-1482.063384	-1482.027221	-1482.133724	-1,482.61377054
**Ag-Int1-β**	-1830.40829323	-1829.688149	-1829.642686	-1829.769679	-1,830.37704143
**Ag-TS1-β**	-1830.35987726	-1829.638620	-1829.593746	-1829.716270	-1,830.32518634
**Ag-Int2-β**	-1830.46424470	-1829.741146	-1829.695738	-1829.820284	-1,830.42965078
**Ag-Int1-α**	-1830.40931420	-1829.687934	-1829.641344	-1829.768633	-1,830.37479072
**Ag-TS1-α**	-1830.35774246	-1829.636854	-1829.591679	-1829.715327	-1,830.32281756
**Ag-Int2-α**	-1830.45377501	-1829.730320	-1829.684891	-1829.81192	-1,830.41603394
**Ag-Int3-β**	-1946.21460483	-1945.437115	-1945.387568	-1945.521641	-1,946.18192594
**Ag-TS2-β**	-1946.18031029	-1945.408780	-1945.359781	-1945.491832	-1,946.14610009
**Ag-Int4-β**	-1946.22883274	-1945.450090	-1945.401523	-1945.531794	-1,946.19457976
**Ag-Int3-α**	-1946.20737641	-1945.429898	-1945.381227	-1945.512954	-1,946.17218631
**Ag-TS2-α**	-1946.17592820	-1945.404165	-1945.355514	-1945.486353	-1,946.14181534
**Ag-Int4-α**	-1946.22413710	-1945.446391	-1945.397291	-1945.530319	-1,946.18921104
**Ag-Int5**	-2009.14426239	-2008.336317	-2008.286374	-2008.419977	-2,009.11170835
**Ag-TS3**	-2009.13430552	-2008.326696	-2008.277666	-2008.406364	-2,009.10208309

[a] Computed at the SMD (solvent=toluene)/B3LYP-D3/6-31G(d)/SDD level.
[b] Computed at the B3LYP-D3/6-31G(d)/SDD level.

Table S2: Singlet-Point Energies (SPEs); Thermal Corrections to Gibbs Energies (TCGs); Corrections Solvent Energies (E_{SMD}) and Free energies $\Delta G_{SMD,DLPNO-CCSD(T)}$ (kcal/mol) for Ag-system.

	SPE[a] (a.u)	TCG (a.u)	ΔE_{SMD} (a.u)	$\Delta G_{SMD,DLPNO-CCSD(T)}$
**substrate**	-347.040470772394	0.102751	-0.011173140	
**B_2(pin)$_2$**	-821.120864401003	0.318362	-0.012880099	
**MeOH**	-115.541678932411	0.028491	-0.002709754	
**product**	-758.261429550398	0.289554	-0.045461577	
**MetOBpin**	-525.568421880140	0.188094	-0.007828880	
**Ag-Catalyst**	-1479.61025750791	0.511061	-0.031014570	0.0
**Ag-Int1-β**	-1826.67514694816	0.638614	-0.031251800	7.1
**Ag-TS1-β**	-1826.64302964388	0.643607	-0.034690920	28.2
**Ag-Int2-β**	-1826.73119142896	0.643961	-0.034593920	-26.8
**Ag-Int1-α**	-1826.67247139725	0.640681	-0.034523480	8.0
**Ag-TS1-α**	-1826.63166809175	0.642416	-0.034924900	34.5
**Ag-Int2-α**	-1826.71971578253	0.641855	-0.037741070	-22.9
**Ag-Int3-β**	-1942.30702232579	0.692964	-0.032678890	-32.5
**Ag-TS2-β**	-1942.27121599064	0.688478	-0.034210200	-13.8

**Ag-Int4-β**	-1942.31983880287	0.697039	-0.034252980	-38.9
**Ag-Int3-α**	-1942.28374135602	0.694422	-0.035190100	-18.5
**Ag-TS2-α**	-1942.25093852340	0.689575	-0.034112860	-0.3
**Ag-Int4-α**	-1942.29310253048	0.693818	-0.034926060	-24.6
**Ag-Int5**	-2005.17982666411	0.724286	-0.032554040	-59.6
**Ag-TS3**	-2005.17241234929	0.727941	-0.032222430	-52.5

[a] Computed at the DLPNO-CCSD(T)/def2-TZVP level.

Table S3: Energies by optimization calculations. Electronic Energy (EE); Sum of electronic and zero-point Energies (SZPE); Sum of electronic and thermal Enthalpies (SE) and Sum of electronic and thermal Free Energies (SFE) for Cu-system.

	EE[a] (a.u)	SZPE[a] (a.u)	SE[a] (a.u)	SFE[a] (a.u)	EE $_{gas\text{-}phase}$[b] (a.u)
**substrate**	-347.739577425	-347.601284	-347.591969	-347.636826	-347.728404285
**$B_2(pin)_2$**	-822.638119861	-822.273177	-822.252883	-822.319758	-822.625239762
**MeOH**	-115.727798327	-115.676557	-115.672291	-115.699308	-115.725088573
**product**	-759.739850806	-759.404662	-759.385912	-759.450297	-759.694389229
**MetOBpin**	-526.506194703	-526.280817	-526.267629	-526.318100	-526.498365823
**Cu-Catalyst**	-1532.99567937	-1532.414579	-1532.379376	-1532.482199	-1532.9626080
**Cu-Int1-β**	-1880.76108939	-1880.038159	-1879.993023	-1880.114714	-1880.7278480
**Cu-TS1-β**	-1880.74722217	-1880.024651	-1879.980294	-1880.101681	-1880.7100160
**Cu-Int2-β**	-1880.82577026	-1880.102222	-1880.057071	-1880.182406	-1880.7879750
**Cu-Int1-α**	-1880.75674239	-1880.034337	-1879.988922	-1880.112615	-1880.7222960
**Cu-TS1-α**	-1880.74334636	-1880.020655	-1879.976364	-1880.095906	-1880.7072660
**Cu-Int2-α**	-1880.81582887	-1880.091480	-1880.046754	-1880.168338	-1880.7755110
**Cu-Int3-β**	-1996.57583137	-1995.797704	-1995.748679	-1995.879654	-1996.5400530
**Cu-TS2-β**	-1996.54213051	-1995.769646	-1995.721153	-1995.851892	-1996.5076070
**Cu-Int4-β**	-1996.59974702	-1995.820911	-1995.772365	-1995.902972	-1996.5645000
**Cu-Int3-α**	-1996.56902531	-1995.790884	-1995.741740	-1995.874253	-1996.5310270
**Cu-TS2-α**	-1996.54022240	-1995.767749	-1995.719495	-1995.848489	-1996.5068180
**Cu-Int4-α**	-1996.59511928	-1995.816554	-1995.767911	-1995.898624	-1996.5592180
**Cu-Int5**	-2059.50757734	-2058.698626	-2058.649316	-2058.779465	-2059.4725240
**Cu-TS3**	-2059.49634902	-2058.689159	-2058.639877	-2058.771827	-2059.4648550

[a] Computed at the SMD (solvent=toluene)/B3LYP-D3/6-31G(d)/SDD level.
[b] Computed at the B3LYP-D3/6-31G(d)/SDD level.

Table S4: Singlet-Point Energies (SPEs); Thermal Corrections to Gibbs Energies (TCGs); Corrections Solvent Energies (E_{SMD}) and Free energies $\Delta G_{SMD,DLPNO\text{-}CCSD(T)}$ (kcal/mol) for Cu-system.

	SPE[a] (a.u)	TCG (a.u)	ΔE_{SMD} (a.u)	$\Delta G_{SMD,DLPNO\text{-}CCSD(T)}$
**substrate**	-347.040470772394	0.102751	-0.011173140	
**$B_2(pin)_2$**	-821.120864401003	0.318362	-0.012880099	
**MeOH**	-115.541678932411	0.028491	-0.002709754	
**product**	-758.261429550398	0.289554	-0.045461577	
**MetOBpin**	-525.568421880140	0.188094	-0.007828880	

**Cu-Catalyst**	-2972.56060364099	0.513480	-0.033071370	0.0
**Cu-Int1-β**	-3319.62946568101	0.646375	-0.033241390	8.0
**Cu-TS1-β**	-3319.61145796407	0.645541	-0.037206170	16.3
**Cu-Int2-β**	-3319.67945402134	0.643364	-0.037795260	-28.1
**Cu-Int1-α**	-3319.62443243353	0.644127	-0.034446390	9.0
**Cu-TS1-α**	-3319.60819305436	0.647440	-0.036080360	20.2
**Cu-Int2-α**	-3319.66704466690	0.647491	-0.040317870	-19.3
**Cu-Int3-β**	-3435.24571637232	0.696177	-0.035778370	-25.3
**Cu-TS2-β**	-3435.21639296199	0.690239	-0.034523510	-9.8
**Cu-Int4-β**	-3435.27223376308	0.696775	-0.035247020	-41.2
**Cu-Int3-α**	-3435.23219161471	0.694772	-0.037998310	-19.1
**Cu-TS2-α**	-3435.20746845552	0.691733	-0.033404400	-2.6
**Cu-Int4-α**	-3435.25083655476	0.696495	-0.035901280	-28.4
**Cu-Int5**	-3498.13682692616	0.728112	-0.035053340	-63.2
**Cu-TS3**	-3498.12950122445	0.724522	-0.031494020	-58.6

[a] Computed at the DLPNO-CCSD(T)/def2-TZVP level.

II. Cartesian Coordinates

Substrate

C	2.607124	0.002218	-0.001011
C	1.394202	0.002805	-0.001090
C	4.064988	-0.001451	0.000444
H	4.459907	-0.361259	0.958001
H	4.461218	-0.652782	-0.787395
H	4.464105	1.005395	-0.166720
C	-0.035711	0.001589	-0.000568
C	-0.750962	-1.212388	-0.000348
C	-0.754032	1.213730	-0.000098
C	-2.143709	-1.209396	0.000318
H	-0.203998	-2.149947	-0.000655
C	-2.146782	1.207105	0.000582
H	-0.209562	2.152739	-0.000200
C	-2.846621	-0.002044	0.000793
H	-2.682192	-2.152744	0.000500
H	-2.687731	2.149036	0.000982
H	-3.932726	-0.003442	0.001360

$B_2(pin)_2$

C	0.232406	-0.750465	3.000353
C	-0.232406	0.750465	3.000353
C	0.750465	0.232406	-3.000353
C	-0.750465	-0.232406	-3.000353
O	0.000000	1.145364	1.612515
O	0.000000	-1.145364	1.612515
O	1.145364	0.000000	-1.612515
O	-1.145364	0.000000	-1.612515
C	0.568017	1.675703	3.909131
H	0.183693	2.696810	3.828935
H	0.479717	1.362484	4.954621
H	1.625204	1.693157	3.638459
C	-1.733395	0.920209	3.253221
H	-1.995133	0.703355	4.292963
H	-2.016793	1.953506	3.034500
H	-2.319109	0.266251	2.600991
C	-0.568017	-1.675703	3.909131
H	-0.183693	-2.696810	3.828935
H	-0.479717	-1.362484	4.954621
H	-1.625204	-1.693157	3.638459
C	1.733395	-0.920209	3.253221
H	1.995133	-0.703355	4.292963
H	2.016793	-1.953506	3.034500
H	2.319109	-0.266251	2.600991
C	-0.920209	-1.733395	-3.253221
H	-1.953506	-2.016793	-3.034500
H	-0.703355	-1.995133	-4.292963
H	-0.266251	-2.319109	-2.600991
C	-1.675703	0.568017	-3.909131
H	-1.362484	0.479717	-4.954621
H	-2.696810	0.183693	-3.828935
H	-1.693157	1.625204	-3.638459
C	1.675703	-0.568017	-3.909131
H	1.362484	-0.479717	-4.954621
H	2.696810	-0.183693	-3.828935
H	1.693157	-1.625204	-3.638459
C	0.920209	1.733395	-3.253221
H	1.953506	2.016793	-3.034500
H	0.703355	1.995133	-4.292963
H	0.266251	2.319109	-2.600991
B	0.000000	0.000000	-0.851814
B	0.000000	0.000000	0.851814

MeOH

C	-0.047142	0.661020	0.000000
H	-1.092405	0.985128	0.000000
H	0.438785	1.087531	0.891640
H	0.438785	1.087531	-0.891640
O	-0.047142	-0.759661	0.000000
H	0.874822	-1.049025	0.000000

Product

C	1.436422000	0.268395000	0.079646000
C	0.525940000	-0.701156000	-0.178628000
C	-2.857161000	0.969621000	0.072391000
C	-3.242573000	-0.555484000	0.025735000
B	-0.976134000	-0.311899000	-0.072157000
O	-1.430215000	0.914588000	0.361299000
O	-1.995973000	-1.172074000	-0.410102000
C	-3.004521000	1.669737000	-1.282500000
H	-4.054769000	1.813947000	-1.552285000
H	-2.525199000	2.651154000	-1.228636000
H	-2.514629000	1.099146000	-2.076749000
C	-3.545179000	1.778023000	1.165863000
H	-3.200610000	2.815685000	1.129797000
H	-4.630749000	1.776361000	1.023802000
H	-3.321514000	1.385248000	2.159079000
C	-4.339640000	-0.915769000	-0.968816000
H	-4.526275000	-1.993417000	-0.939171000
H	-5.273688000	-0.403218000	-0.716733000
H	-4.061217000	-0.651791000	-1.990595000
C	-3.558383000	-1.140501000	1.406102000
H	-4.520581000	-0.785306000	1.786326000
H	-3.599373000	-2.230487000	1.328694000
H	-2.779955000	-0.882567000	2.129794000
H	1.035929000	1.258889000	0.295878000
C	0.851194000	-2.118437000	-0.594410000

H	0.899088000	-2.793038000	0.271199000
H	0.063763000	-2.512416000	-1.244280000
H	1.805361000	-2.195074000	-1.124418000
C	2.906533000	0.214964000	0.078104000
C	3.649383000	-0.938120000	0.398328000
C	3.618437000	1.393479000	-0.222791000
C	5.043407000	-0.917187000	0.385723000
H	3.134962000	-1.845402000	0.690971000
C	5.010426000	1.411745000	-0.244615000
H	3.063424000	2.300178000	-0.450192000
C	5.730612000	0.252788000	0.056451000
H	5.595153000	-1.817349000	0.642217000
H	5.534958000	2.331223000	-0.489099000
H	6.816709000	0.265035000	0.046114000

MetOB(pin)

C	1.072259	-0.691311	-0.014478
C	0.715436	0.843825	0.034167
O	-0.178396	-1.285317	-0.463303
O	-0.709980	0.815939	0.336393
C	0.864011	1.537351	-1.323580
H	0.401828	2.527012	-1.269069
H	1.914078	1.663367	-1.602532
H	0.359346	0.971496	-2.112165
C	1.428942	1.639529	1.120101
H	2.513030	1.613388	0.969933
H	1.107596	2.684731	1.084305
H	1.204011	1.253704	2.115784
C	2.171256	-1.065009	-1.001922
H	3.110751	-0.566172	-0.742745
H	2.342387	-2.145205	-0.973125
H	1.902507	-0.795838	-2.024949
C	1.372448	-1.279757	1.367969
H	1.394293	-2.370297	1.291830
H	2.338901	-0.940831	1.752277
H	0.596068	-1.007499	2.088946
B	-1.174159	-0.406768	-0.102914
O	-2.486609	-0.742386	-0.187467
C	-3.471891	0.242054	0.124104
H	-4.444264	-0.153214	-0.181574
H	-3.498136	0.450076	1.200471
H	-3.286495	1.183149	-0.405979

Ag-Catalyst

C	-1.343994000	1.210195000	0.038456000
C	-2.495691000	3.160269000	0.286854000
C	-3.403537000	2.150952000	0.291203000
N	-1.247438000	2.564232000	0.131833000
N	-2.680465000	0.971613000	0.137832000
B	1.851794000	-1.662223000	-0.094849000
O	3.151801000	-1.406647000	-0.527530000
O	1.742646000	-2.986600000	0.325805000
C	3.995209000	-2.536697000	-0.174346000
C	2.952872000	-3.699540000	-0.048893000
C	3.263738000	-4.738808000	1.024444000
H	2.477373000	-5.500266000	1.039390000
H	4.216130000	-5.240932000	0.819934000
H	3.312379000	-4.289802000	2.018370000
C	2.661265000	-4.396043000	-1.384528000
H	3.501237000	-5.015416000	-1.715889000
H	1.785024000	-5.039892000	-1.263800000
H	2.434247000	-3.663676000	-2.164159000
C	5.048799000	-2.724816000	-1.261598000
H	5.721224000	-1.861126000	-1.278604000
H	5.651969000	-3.619357000	-1.070002000
H	4.595659000	-2.814241000	-2.250751000
C	4.671349000	-2.185499000	1.157189000
H	5.381435000	-2.957279000	1.472001000
H	5.214910000	-1.243274000	1.040146000
H	3.927733000	-2.047488000	1.947067000
Ag	0.266890000	-0.239693000	-0.067809000
H	-4.477638000	2.156122000	0.388864000
H	-2.614148000	4.228170000	0.379617000
C	-3.262361000	-0.344959000	0.091555000
C	-3.284375000	-1.118713000	1.261732000
C	-3.774948000	-0.812956000	-1.128134000
C	-3.851119000	-2.395039000	1.186829000
C	-4.332488000	-2.094599000	-1.151334000
C	-4.375946000	-2.900241000	-0.007538000
H	-3.877425000	-3.009948000	2.083185000
H	-4.734312000	-2.475622000	-2.087189000
C	0.002249000	3.277825000	0.084938000
C	0.443609000	3.786588000	-1.145689000
C	0.743421000	3.420147000	1.266945000
C	1.663888000	4.467774000	-1.168347000
C	1.957968000	4.109149000	1.193119000
C	2.434308000	4.636698000	-0.012070000
H	2.023389000	4.869861000	-2.112600000
H	2.547338000	4.230607000	2.098743000
C	-2.690490000	-0.604756000	2.548682000
C	-4.944709000	-4.296984000	-0.069600000
C	-3.703428000	0.031614000	-2.375869000
C	-0.359362000	3.574849000	-2.404221000
C	3.766058000	5.344531000	-0.072587000
C	0.261985000	2.819984000	2.563426000
H	-2.972179000	-1.241293000	3.391583000
H	-1.596026000	-0.592499000	2.486685000
H	-3.013442000	0.418545000	2.767250000
H	-5.372943000	-4.600500000	0.890862000
H	-5.725204000	-4.380459000	-0.832379000
H	-4.162204000	-5.023751000	-0.321522000
H	-2.662382000	0.248320000	-2.640500000
H	-4.168067000	-0.480760000	-3.222225000
H	-4.206744000	0.996052000	-2.245529000
H	0.323337000	1.725970000	2.525516000
H	0.871122000	3.163287000	3.403709000

H	-0.782925000	3.076011000	2.769008000
H	3.775191000	6.117859000	-0.847242000
H	4.015507000	5.816749000	0.882704000
H	4.571968000	4.638334000	-0.308235000
H	0.106332000	4.078835000	-3.254900000
H	-0.433203000	2.507304000	-2.640640000
H	-1.383308000	3.951739000	-2.304785000

Ag-Int1-β

C	0.580066	1.507696	-1.006217
C	2.131362	2.689917	-2.187574
C	1.126904	3.545695	-1.871640
N	1.781013	1.454834	-1.649373
N	0.193295	2.805218	-1.152227
B	-2.060302	-1.689764	0.123706
O	-3.141673	-1.993448	-0.705547
O	-2.024542	-2.586233	1.193011
C	-3.713512	-3.264662	-0.295313
C	-3.250562	-3.368111	1.198215
C	-2.930020	-4.778603	1.685022
H	-2.579372	-4.740443	2.721313
H	-3.820232	-5.416721	1.651655
H	-2.146623	-5.246568	1.085379
C	-4.211593	-2.680017	2.175938
H	-5.148952	-3.234773	2.286829
H	-3.732499	-2.612596	3.157162
H	-4.440908	-1.663433	1.843881
C	-5.224589	-3.214534	-0.505212
H	-5.447051	-3.126086	-1.573508
H	-5.703899	-4.128046	-0.135348
H	-5.672093	-2.356690	0.000692
C	-3.093328	-4.341138	-1.196677
H	-3.511152	-5.333478	-0.997526
H	-3.294074	-4.086397	-2.241643
H	-2.008089	-4.383727	-1.065356
Ag	-0.671466	-0.126395	-0.279833
H	0.986143	4.592910	-2.087410
H	3.048449	2.837714	-2.735436
C	1.997993	-1.840018	1.726308
C	1.564526	-2.736592	1.034551
C	-1.026033	3.340208	-0.604827
C	-0.973106	3.984987	0.639608
C	-2.223719	3.183855	-1.320196
C	-2.167164	4.490962	1.164098
C	-3.389856	3.706848	-0.754199
C	-3.382751	4.358455	0.484826
H	-2.145789	4.994558	2.127475
H	-4.328187	3.597334	-1.292699
C	2.595440	0.269558	-1.717792
C	2.230672	-0.760938	-2.595650
C	3.711405	0.174305	-0.868441
C	3.004351	-1.928289	-2.583640
C	4.452691	-1.008265	-0.899744
C	4.105765	-2.075539	-1.736495
H	2.731341	-2.742803	-3.250393
H	5.310257	-1.106678	-0.238730
C	-2.259831	2.450845	-2.636945
C	0.328396	4.109427	1.391413
C	-4.667187	4.889063	1.074359
C	1.031899	-0.646404	-3.504062
C	4.078082	1.294423	0.072524
C	4.882020	-3.368482	-1.687611
H	0.181255	4.619657	2.346777
H	0.762023	3.124437	1.592602
H	1.075902	4.671840	0.820537
H	-5.254278	5.433808	0.326888
H	-5.297023	4.070154	1.442797
H	-4.477784	5.563516	1.914597
H	-1.428302	2.737233	-3.288725
H	-2.185319	1.369219	-2.472432
H	-3.195680	2.645193	-3.167876
H	0.105980	-0.847644	-2.951533
H	0.937236	0.355549	-3.933902
H	1.096970	-1.367726	-4.323502
H	4.957785	1.032945	0.663634
H	4.294862	2.224742	-0.463675
H	3.262786	1.505284	0.770712
H	4.581979	-3.962684	-0.815147
H	4.708573	-3.979604	-2.578411
H	5.959125	-3.189901	-1.601270
C	0.992550	-3.788156	0.205931
H	-0.064043	-3.915039	0.461812
H	1.051718	-3.517767	-0.854691
H	1.512646	-4.743370	0.345122
C	2.468122	-0.746577	2.514484
C	1.594096	0.307832	2.853526
C	3.806047	-0.687347	2.949953
C	2.055679	1.391463	3.598848
H	0.562373	0.262927	2.517253
C	4.258625	0.400828	3.694601
H	4.481738	-1.497539	2.693233
C	3.388561	1.444921	4.019422
H	1.371572	2.195919	3.854067
H	5.294353	0.434194	4.021428
H	3.744265	2.291360	4.600159

Ag-TS1-β

C	0.017848	-1.862938	0.095704
C	0.774971	-4.002081	0.285247
C	-0.520465	-4.064782	-0.113109
N	1.086240	-2.652448	0.405468
N	-0.968101	-2.749470	-0.219912
B	-1.294753	1.839956	0.399339
O	-1.629573	2.206740	1.684110

O	-2.242527	2.232794	-0.522435
C	-2.875651	2.971874	1.641251
C	-3.441392	2.625847	0.210620
C	-4.096974	3.790825	-0.523817
H	-4.430526	3.459266	-1.511843
H	-4.973064	4.151911	0.025186
H	-3.407211	4.624603	-0.666156
C	-4.370770	1.408270	0.198633
H	-5.328399	1.622486	0.683706
H	-4.560411	1.122544	-0.838578
H	-3.906520	0.552353	0.692391
C	-3.751058	2.510358	2.803322
H	-3.272090	2.772787	3.751499
H	-4.730444	2.999668	2.772499
H	-3.899884	1.429203	2.790898
C	-2.512693	4.448749	1.823214
H	-3.409006	5.072702	1.898638
H	-1.939042	4.561490	2.747800
H	-1.900726	4.820379	1.000059
Ag	0.113065	0.212011	-0.031992
H	-1.160964	-4.903726	-0.333673
H	1.498619	-4.775718	0.486465
C	1.700681	1.591057	-0.811150
C	0.720353	2.292798	-0.309781
C	-2.307593	-2.386052	-0.598398
C	-2.551653	-1.916635	-1.896111
C	-3.333519	-2.527537	0.353342
C	-3.872170	-1.591458	-2.232979
C	-4.633237	-2.197437	-0.034299
C	-4.921621	-1.726568	-1.321809
H	-4.079095	-1.223233	-3.234783
H	-5.439130	-2.296914	0.689231
C	2.377787	-2.129972	0.774307
C	2.671378	-1.964856	2.134408
C	3.273280	-1.756895	-0.241419
C	3.913678	-1.414799	2.470162
C	4.493969	-1.199796	0.143425
C	4.829805	-1.019507	1.490693
H	4.160960	-1.277890	3.520109
H	5.186701	-0.871669	-0.626838
C	-3.035999	-2.990435	1.758155
C	-1.440043	-1.742458	-2.899938
C	-6.332672	-1.337074	-1.688941
C	1.665899	-2.337317	3.195425
C	2.909907	-1.893728	-1.697072
C	6.146937	-0.383290	1.858396
H	-1.837588	-1.711693	-3.918029
H	-0.900443	-0.804705	-2.723848
H	-0.704195	-2.550175	-2.842148
H	-7.046540	-2.127076	-1.430439
H	-6.640389	-0.435535	-1.145393
H	-6.429943	-1.129439	-2.758276
H	-2.590618	-3.990850	1.776262
H	-2.325391	-2.316542	2.249319
H	-3.948134	-3.018037	2.359573
H	0.767450	-1.714983	3.115053
H	1.341085	-3.379693	3.103092
H	2.083409	-2.200144	4.196277
H	3.790770	-1.774347	-2.331742
H	2.451115	-2.863493	-1.917041
H	2.189035	-1.114840	-1.969337
H	6.193399	0.643020	1.476701
H	6.296019	-0.351681	2.941648
H	6.990084	-0.926708	1.416450
C	0.531616	3.767328	-0.070646
H	-0.312245	4.168721	-0.641299
H	0.357166	3.988409	0.986894
H	1.443624	4.285932	-0.390814
C	3.054496	1.840313	-1.236025
C	3.485688	1.556243	-2.552779
C	4.030069	2.300048	-0.316688
C	4.814490	1.735276	-2.930709
H	2.760237	1.186712	-3.272587
C	5.359027	2.466270	-0.699961
H	3.727034	2.510678	0.705122
C	5.766587	2.184326	-2.008620
H	5.112387	1.514500	-3.953246
H	6.082853	2.826809	0.027698
H	6.803854	2.313986	-2.304185

Ag-Int2-β

C	0.274145	-1.991721	0.090135
C	1.145637	-4.069359	0.430346
C	-0.171865	-4.224182	0.144523
N	1.397242	-2.702860	0.394246
N	-0.685597	-2.944913	-0.058554
B	-1.399813	2.957565	0.088429
O	-2.078696	3.869839	0.876282
O	-2.172273	1.840394	-0.184208
C	-3.451473	3.418662	1.003802
C	-3.330248	1.881365	0.687922
C	-4.518929	1.276807	-0.051243
H	-4.320895	0.221469	-0.247475
H	-5.431282	1.347995	0.551148
H	-4.692249	1.771002	-1.009012
C	-2.985141	1.030636	1.916153
H	-3.822138	0.970292	2.618644
H	-2.739653	0.018578	1.582502
H	-2.113845	1.435603	2.439165
C	-3.947716	3.750532	2.407490
H	-3.986217	4.836116	2.540028
H	-4.955677	3.353611	2.568782
H	-3.285182	3.343682	3.173935
C	-4.264352	4.187891	-0.043922
H	-5.333238	3.962514	0.020756

H	-4.127677	5.260670	0.120218
H	-3.918481	3.956173	-1.055429
Ag	0.338391	0.105157	-0.240170
H	-0.786369	-5.106395	0.060937
H	1.918902	-4.788199	0.650073
C	0.871718	2.124522	-0.591728
C	0.051695	3.195611	-0.397450
C	-2.060047	-2.682442	-0.394250
C	-2.395730	-2.399459	-1.727321
C	-3.023763	-2.747608	0.624286
C	-3.746559	-2.197757	-2.028010
C	-4.361243	-2.542921	0.271492
C	-4.741805	-2.272589	-1.047633
H	-4.026467	-1.976137	-3.054990
H	-5.121406	-2.586565	1.047520
C	2.677168	-2.088462	0.647791
C	3.039183	-1.814010	1.974079
C	3.491010	-1.736937	-0.441801
C	4.258215	-1.163850	2.195079
C	4.694039	-1.083392	-0.169350
C	5.090716	-0.782373	1.138571
H	4.553320	-0.937305	3.216619
H	5.323444	-0.776346	-0.999694
C	-2.628870	-3.011262	2.056298
C	-1.340396	-2.289320	-2.798395
C	-6.184646	-2.003286	-1.396173
C	2.125110	-2.164607	3.121334
C	3.059204	-2.000629	-1.861538
C	6.378477	-0.036480	1.383516
H	-1.789441	-2.334549	-3.793958
H	-0.804327	-1.336886	-2.712102
H	-0.593630	-3.086036	-2.721175
H	-6.867235	-2.467049	-0.677714
H	-6.386436	-0.924462	-1.392214
H	-6.434121	-2.375762	-2.394715
H	-2.245221	-4.028650	2.193816
H	-1.837830	-2.326989	2.380187
H	-3.483561	-2.882496	2.725031
H	1.209526	-1.562484	3.086706
H	1.817377	-3.215431	3.096510
H	2.613679	-1.977307	4.081015
H	3.896288	-1.875158	-2.552775
H	2.656118	-3.010794	-1.987752
H	2.273244	-1.296580	-2.157284
H	6.300607	0.987207	0.999784
H	6.620661	0.018376	2.448774
H	7.220488	-0.511916	0.867669
C	0.465747	4.653656	-0.556333
H	-0.244516	5.195991	-1.194691
H	0.456564	5.175215	0.410294
H	1.462909	4.769922	-0.989518
C	2.310279	2.286556	-0.925600
C	2.848729	1.740966	-2.109207
C	3.207535	2.918777	-0.040409
C	4.207719	1.838291	-2.403560
H	2.179636	1.227096	-2.795112
C	4.570810	3.006213	-0.327091
H	2.819692	3.338162	0.884082
C	5.081862	2.469279	-1.511845
H	4.589148	1.413224	-3.329228
H	5.236172	3.501926	0.376198
H	6.142940	2.538319	-1.735883

Ag-Int1-α

C	1.700319	-1.413817	-0.377248
C	2.399155	-3.558563	-0.708468
C	3.508738	-2.787508	-0.583612
N	1.308902	-2.701965	-0.575933
N	3.061652	-1.485255	-0.381632
B	-0.764416	2.108380	-0.536676
O	-1.601493	2.421413	-1.606941
O	-0.915086	3.053292	0.475481
C	-2.521346	3.469549	-1.195894
C	-1.754846	4.137963	-0.000925
C	-2.639644	4.606742	1.151453
H	-2.017731	5.040322	1.941106
H	-3.347750	5.374124	0.818723
H	-3.200877	3.777403	1.586243
C	-0.813748	5.267846	-0.437574
H	-1.362003	6.158154	-0.763048
H	-0.177155	5.546272	0.407743
H	-0.161628	4.939474	-1.251871
C	-2.785594	4.383078	-2.389694
H	-3.304922	3.823158	-3.174116
H	-3.419341	5.230394	-2.104548
H	-1.857449	4.769501	-2.815581
C	-3.818031	2.774637	-0.765228
H	-4.599007	3.494926	-0.499573
H	-4.183645	2.161076	-1.593277
H	-3.642718	2.114171	0.085715
Ag	0.464191	0.376709	-0.394516
H	4.557798	-3.034723	-0.620689
H	2.279312	-4.617045	-0.877164
C	-0.562787	-0.071852	3.002345
C	-1.653105	-0.134585	2.474518
C	3.918217	-0.353724	-0.138396
C	4.468025	-0.198150	1.144235
C	1.700319	-1.413817	-0.377248
C	4.153551	0.567292	-1.171455
C	5.266923	0.924211	1.381867
C	4.956945	1.674744	-0.881863
C	5.514955	1.874604	0.385512
H	5.696311	1.062625	2.371135

H	5.146686	2.402116	-1.667600
C	-0.065254	-3.131058	-0.618185
C	-0.782630	-3.000018	-1.816035
C	-0.640079	-3.667689	0.546500
C	-2.114131	-3.433963	-1.828339
C	-1.968513	-4.093459	0.479585
C	-2.720913	-3.984507	-0.695854
H	-2.686806	-3.336934	-2.747476
H	-2.434045	-4.502154	1.373139
C	3.542880	0.393612	-2.539189
C	4.200056	-1.204175	2.236724
C	6.338293	3.105755	0.676516
C	-0.159098	-2.379598	-3.040627
C	0.121830	-3.719015	1.846993
C	-4.169001	-4.406192	-0.711770
H	4.521013	-0.818364	3.207851
H	3.136188	-1.449751	2.301654
H	4.734637	-2.145180	2.059735
H	5.690071	3.956757	0.920123
H	7.009786	2.951103	1.526460
H	6.944415	3.397782	-0.187280
H	3.587399	-0.646549	-2.876746
H	2.486092	0.686922	-2.528804
H	4.057472	1.016115	-3.276219
H	-0.079938	-1.292123	-2.921949
H	0.852185	-2.758105	-3.220670
H	-0.763939	-2.577955	-3.929322
H	-0.388045	-4.359244	2.571517
H	1.143000	-4.090724	1.719973
H	0.187963	-2.714346	2.279950
H	-4.780617	-3.692586	-0.146760
H	-4.565986	-4.452260	-1.730086
H	-4.306392	-5.389174	-0.248221
C	-2.918664	-0.184616	1.812798
C	-4.073249	0.350466	2.418038
C	-3.023528	-0.754343	0.528426
C	-5.299336	0.309414	1.756013
H	-3.995626	0.799877	3.403327
C	-4.253780	-0.792567	-0.124103
H	-2.137792	-1.155598	0.051507
C	-5.395409	-0.264430	0.484922
H	-6.181034	0.729210	2.232174
H	-4.313423	-1.230140	-1.116042
H	-6.351454	-0.290750	-0.030300
C	0.771627	0.049173	3.576205
H	1.185049	-0.928999	3.849813
H	1.450175	0.514657	2.851654
H	0.764654	0.670703	4.478909

Ag-TS1-α

C	1.987035	0.823061	0.152685
C	3.397401	2.320295	1.129739
C	3.983559	1.100054	1.212341
N	2.179500	2.132112	0.479344
N	3.108980	0.196878	0.610501
B	-1.405788	-0.457317	0.461740
O	-2.283038	0.544505	0.806334
O	-1.500158	-1.549488	1.297876
C	-3.184171	0.043932	1.841282
C	-2.413227	-1.221361	2.390993
C	-3.290022	-2.442256	2.655017
H	-2.665512	-3.274245	2.995061
H	-4.027872	-2.231958	3.436479
H	-3.813661	-2.761964	1.753429
C	-1.544764	-0.927258	3.618773
H	-2.155127	-0.700428	4.498336
H	-0.934213	-1.805791	3.843962
H	-0.869657	-0.087720	3.436781
C	-3.397494	1.160223	2.860180
H	-3.900440	2.003011	2.376398
H	-4.029077	0.818698	3.686951
H	-2.454207	1.525185	3.268583
C	-4.511677	-0.289546	1.154915
H	-5.266898	-0.604688	1.881943
H	-4.879276	0.604581	0.643666
H	-4.395911	-1.074680	0.408170
Ag	0.347729	-0.014879	-0.768601
H	4.924749	0.785705	1.634435
H	3.720322	3.293348	1.464063
C	-0.573380	-0.528556	-2.703960
C	-1.386691	-0.877067	-1.731997
C	3.359817	-1.211050	0.454299
C	4.131186	-1.637932	-0.638051
C	2.804983	-2.110842	1.377648
C	4.356345	-3.010136	-0.781764
C	3.056636	-3.473511	1.188965
C	3.829939	-3.941098	0.120706
H	4.949379	-3.359059	-1.623664
H	2.634871	-4.186536	1.893447
C	1.234251	3.173625	0.179962
C	0.265369	3.500548	1.139478
C	1.294696	3.803157	-1.071642
C	-0.646226	4.512538	0.827986
C	0.359497	4.808247	-1.339034
C	-0.614759	5.175761	-0.404245
H	-1.405001	4.779898	1.559380
H	0.390047	5.311123	-2.302751
C	1.924143	-1.635635	2.504605
C	4.662942	-0.651326	-1.646799
C	4.114034	-5.415466	-0.033740
C	0.182089	2.737918	2.436293
C	2.305249	3.374780	-2.104884
C	-1.634480	6.240400	-0.729142
H	5.320959	-1.144841	-2.366619

H	3.839211	-0.188498	-2.201981
H	5.225140	0.159125	-1.171116
H	3.247839	-6.022246	0.249045
H	4.382125	-5.667382	-1.064116
H	4.949559	-5.723319	0.608033
H	2.302436	-0.716273	2.962272
H	0.916132	-1.427804	2.131735
H	1.845076	-2.398904	3.283882
H	-0.135776	1.710175	2.233565
H	1.148478	2.687358	2.949008
H	-0.542792	3.194145	3.114987
H	2.324400	4.070659	-2.947697
H	3.316106	3.312123	-1.688130
H	2.051490	2.379499	-2.488328
H	-2.540549	5.794870	-1.158577
H	-1.938156	6.792462	0.166112
H	-1.247948	6.959637	-1.457947
C	-2.695976	-1.554790	-1.707749
C	-2.881462	-2.811124	-1.103731
C	-3.787680	-0.957605	-2.364089
C	-4.119715	-3.450296	-1.159541
H	-2.051200	-3.266212	-0.575408
C	-5.026748	-1.597832	-2.415472
H	-3.652778	0.016734	-2.823854
C	-5.199491	-2.845156	-1.811103
H	-4.243428	-4.423004	-0.690402
H	-5.859756	-1.117924	-2.922490
H	-6.165168	-3.341826	-1.846528
C	-0.708364	-0.766679	-4.176668
H	-0.699982	0.185739	-4.721883
H	0.148980	-1.337182	-4.554638
H	-1.628079	-1.306835	-4.454168

Ag-Int2-α

C	2.332517	0.278244	-0.121195
C	4.210129	1.518382	0.221832
C	4.514791	0.231679	0.527983
N	2.873975	1.526073	-0.170187
N	3.356662	-0.507518	0.311801
B	-2.586866	0.015987	0.430631
O	-2.417503	1.363857	0.174415
O	-2.787569	-0.229386	1.778836
C	-2.766830	2.086668	1.385785
C	-2.520567	1.004933	2.496485
C	-3.452917	1.084466	3.699972
H	-3.212331	0.284048	4.406402
H	-3.337760	2.041664	4.219758
H	-4.497893	0.968335	3.406694
C	-1.058431	0.940653	2.954929
H	-0.781266	1.812406	3.555468
H	-0.917084	0.042091	3.562395
H	-0.389310	0.875106	2.092523
C	-1.890428	3.328267	1.487208
H	-2.126918	4.015292	0.669517
H	-2.063011	3.853033	2.433373
H	-0.833822	3.075317	1.409886
C	-4.242715	2.478356	1.249836
H	-4.583856	3.081129	2.097089
H	-4.370616	3.064251	0.335193
H	-4.877704	1.590976	1.171234
Ag	0.411189	-0.466842	-0.649845
H	5.429497	-0.223347	0.873153
H	4.804789	2.417656	0.241265
C	-1.382663	-1.424648	-1.213552
C	-2.584339	-1.094007	-0.662277
C	3.237268	-1.931311	0.498939
C	3.600012	-2.780896	-0.556983
C	2.720465	-2.414343	1.710285
C	3.447379	-4.157313	-0.366039
C	2.583539	-3.798651	1.851663
C	2.941794	-4.683416	0.828386
H	3.718668	-4.832727	-1.173651
H	2.177700	-4.193620	2.779790
C	2.152149	2.706850	-0.565843
C	1.730669	3.600006	0.431091
C	1.896100	2.925962	-1.927987
C	1.033343	4.742938	0.029650
C	1.186140	4.079442	-2.276928
C	0.745041	4.995118	-1.315953
H	0.691394	5.442552	0.788289
H	0.973053	4.265529	-3.326760
C	2.274774	-1.471884	2.799029
C	4.090410	-2.222619	-1.868983
C	2.810190	-6.174714	1.020804
C	1.996516	3.324020	1.890630
C	2.351188	1.948151	-2.981602
C	-0.058177	6.207856	-1.717515
H	4.406373	-3.023661	-2.542020
H	3.295605	-1.656039	-2.367975
H	4.934872	-1.538532	-1.733485
H	1.962953	-6.420942	1.668177
H	2.670190	-6.692090	0.066883
H	3.710793	-6.590922	1.490416
H	3.007959	-0.679200	2.981075
H	1.334110	-0.984355	2.518416
H	2.108844	-2.007392	3.737446
H	1.622186	2.336903	2.180979
H	3.067578	3.338105	2.121575
H	1.509295	4.070134	2.523102
H	2.295338	2.395695	-3.977308
H	3.380739	1.616961	-2.810838
H	1.720014	1.051891	-2.979165
H	-1.129404	5.971664	-1.743034

H	0.077538	7.033249	-1.011679
H	0.219809	6.562587	-2.714906
C	-3.904554	-1.723370	-0.970612
C	-4.774775	-2.110958	0.068697
C	-4.356676	-1.893732	-2.293478
C	-6.023087	-2.671159	-0.203535
H	-4.457507	-1.970301	1.097952
C	-5.605180	-2.453771	-2.567167
H	-3.721718	-1.566011	-3.111141
C	-6.445053	-2.850289	-1.523577
H	-6.669394	-2.969185	0.618714
H	-5.926196	-2.572780	-3.599257
H	-7.418076	-3.285216	-1.736046
C	-1.233098	-2.576762	-2.195417
H	-1.000532	-2.199431	-3.202034
H	-0.382896	-3.210594	-1.912362
H	-2.116987	-3.223135	-2.280199

Ag-Int3-β

C	-0.664547	2.180956	0.217779
C	0.253913	3.126002	-0.136309
Ag	-0.441532	0.077780	0.011583
C	-0.620182	-2.031125	-0.076992
N	0.238534	-3.042386	0.225440
N	-1.804388	-2.664475	-0.306652
C	-0.397006	-4.280897	0.187307
H	0.126930	-5.197723	0.405538
C	-1.689278	-4.041124	-0.149468
H	-2.526965	-4.706129	-0.287277
C	3.402423	1.375394	-1.319743
C	3.714720	2.887201	-1.630400
B	1.634953	2.690688	-0.688795
O	2.246703	1.473882	-0.447456
O	2.413062	3.510517	-1.483105
C	2.953889	0.576791	-2.548823
H	3.778775	0.402252	-3.246380
H	2.571305	-0.392105	-2.214813
H	2.147734	1.092273	-3.079143
C	4.509406	0.631346	-0.581383
H	4.200081	-0.400376	-0.406057
H	5.430016	0.616399	-1.174924
H	4.724271	1.088926	0.386098
C	4.234253	3.161037	-3.037842
H	4.400295	4.234657	-3.168471
H	5.187277	2.648689	-3.208030
H	3.522036	2.837293	-3.799171
C	4.631156	3.539659	-0.588721
H	5.658228	3.169186	-0.658953
H	4.641517	4.620935	-0.752935
H	4.262119	3.358385	0.424983
O	0.903289	1.296903	2.847335
C	2.318313	1.205037	2.750576
H	2.638508	0.516763	1.960825
H	2.786862	2.184491	2.565485
H	2.688315	0.836648	3.713825
H	0.560682	1.555324	1.971800
C	1.628444	-2.870399	0.557242
C	2.586486	-3.138578	-0.435776
C	1.981052	-2.466253	1.852635
C	3.934048	-3.009135	-0.093955
C	3.346815	-2.341968	2.141102
C	4.332712	-2.609112	1.188782
H	4.690397	-3.210291	-0.848760
H	3.640786	-2.026526	3.138983
C	-3.026395	-1.976843	-0.638835
C	-3.355744	-1.804846	-1.992897
C	-3.828568	-1.484329	0.399546
C	-4.536658	-1.122070	-2.293009
C	-5.002849	-0.809668	0.047405
C	-5.370937	-0.617331	-1.286715
H	-4.809460	-0.975165	-3.335493
H	-5.630084	-0.407180	0.837707
C	2.169959	-3.528019	-1.832314
C	5.794401	-2.416857	1.507766
C	0.947443	-2.139839	2.900148
C	-2.442946	-2.311706	-3.081220
C	-6.617893	0.154244	-1.645033
C	-3.414171	-1.617681	1.842638
H	1.494985	-2.781110	-2.264138
H	3.039987	-3.618601	-2.487428
H	1.634799	-4.484177	-1.848875
H	6.137385	-1.432988	1.162417
H	5.984289	-2.470268	2.583821
H	6.416940	-3.169045	1.012586
H	0.635665	-1.090970	2.822570
H	0.053054	-2.761127	2.802697
H	1.358560	-2.285873	3.903415
H	-1.486949	-1.775504	-3.066845
H	-2.213163	-3.375764	-2.958708
H	-2.893738	-2.172312	-4.067046
H	-7.255490	0.317981	-0.771714
H	-6.362552	1.136972	-2.060018
H	-7.209878	-0.368816	-2.404205
H	-4.240458	-1.363114	2.510971
H	-3.076363	-2.631252	2.082142
H	-2.583163	-0.937175	2.063856
C	0.008627	4.628171	-0.086656
H	-1.035385	4.882741	0.113893
H	0.621511	5.105267	0.691068
H	0.304648	5.096734	-1.033004
C	-1.987016	2.574292	0.771236
C	-3.181457	2.295043	0.079186
C	-2.091855	3.189332	2.035018

C	-4.422697	2.639753	0.612874
H	-3.123298	1.788736	-0.880697
C	-3.335593	3.511911	2.579858
H	-1.182431	3.397907	2.592098
C	-4.509523	3.246227	1.869783
H	-5.327061	2.421973	0.050275
H	-3.386608	3.980137	3.560051
H	-5.477608	3.503722	2.291121

Ag-TS2-β

C	-1.434395	1.851633	1.274477
C	-1.291625	2.419663	0.034572
Ag	-0.058725	-0.125157	0.753951
C	0.283605	-1.824040	-0.449218
N	1.410414	-2.581755	-0.565382
N	-0.619674	-2.449384	-1.252214
C	1.211839	-3.659787	-1.423045
H	1.992724	-4.372145	-1.635197
C	-0.070520	-3.575655	-1.857803
H	-0.639568	-4.197497	-2.530303
C	2.410390	2.947100	-0.612260
C	1.780227	3.207928	-2.033054
B	0.133780	2.701801	-0.515368
O	1.245794	2.978889	0.252260
O	0.425757	2.708398	-1.869966
C	3.031124	1.555024	-0.468062
H	3.959479	1.462389	-1.038621
H	3.251103	1.369108	0.585753
H	2.339042	0.778645	-0.800355
C	3.394191	4.012078	-0.138389
H	3.771335	3.747872	0.854066
H	4.249729	4.082599	-0.818315
H	2.921893	4.993455	-0.066812
C	2.442394	2.449895	-3.178846
H	1.925065	2.672076	-4.117270
H	3.489944	2.748682	-3.290399
H	2.403559	1.370319	-3.021540
C	1.658750	4.696267	-2.378051
H	2.634814	5.149838	-2.574857
H	1.040584	4.804182	-3.273991
H	1.174361	5.247357	-1.566947
O	0.404124	0.879883	2.851176
C	1.608507	1.523253	3.210956
H	1.848181	2.351150	2.529480
H	1.556707	1.930561	4.235531
H	2.446832	0.809608	3.191871
H	-0.341950	1.522021	2.249873
C	2.663481	-2.272419	0.072975
C	3.708993	-1.773196	-0.721701
C	2.800066	-2.457426	1.458982
C	4.916602	-1.457394	-0.092342
C	4.026914	-2.118843	2.039627
C	5.094109	-1.620172	1.285425
H	5.732084	-1.061108	-0.692230
H	4.149498	-2.250155	3.112060
C	-1.971683	-1.995264	-1.458382
C	-2.203604	-0.941104	-2.356878
C	-3.008417	-2.625688	-0.752960
C	-3.526082	-0.521094	-2.533525
C	-4.313819	-2.177788	-0.977260
C	-4.590744	-1.122054	-1.852668
H	-3.727007	0.300898	-3.215821
H	-5.129628	-2.650558	-0.436491
C	3.536535	-1.556274	-2.205359
C	6.409747	-1.288496	1.946534
C	1.674579	-2.976315	2.319119
C	-1.072653	-0.254991	-3.080035
C	-5.995045	-0.593201	-2.003984
C	-2.722769	-3.713220	0.252207
H	2.593544	-1.048155	-2.428779
H	4.352710	-0.949673	-2.605406
H	3.527108	-2.503073	-2.757956
H	6.257574	-0.769549	2.898598
H	6.981551	-2.199622	2.163047
H	7.032232	-0.652933	1.309852
H	1.021900	-2.156095	2.640274
H	1.051163	-3.701160	1.788321
H	2.069465	-3.456992	3.218585
H	-0.549528	0.446921	-2.421786
H	-0.333748	-0.973080	-3.450372
H	-1.448268	0.316021	-3.933154
H	-6.743192	-1.356950	-1.770942
H	-6.157564	0.247600	-1.318103
H	-6.182870	-0.227148	-3.018271
H	-3.651606	-4.132361	0.646782
H	-2.136086	-4.531562	-0.177898
H	-2.149176	-3.316409	1.097691
C	-2.437049	2.796566	-0.896342
H	-3.416965	2.700421	-0.422038
H	-2.331054	3.827733	-1.258340
H	-2.436563	2.159964	-1.790657
C	-2.765035	1.497609	1.819679
C	-3.598527	0.544529	1.198434
C	-3.202816	2.046383	3.040889
C	-4.814729	0.164977	1.765873
H	-3.276442	0.098808	0.263495
C	-4.429306	1.683855	3.597655
H	-2.566401	2.766051	3.549864
C	-5.242862	0.736826	2.967261
H	-5.429107	-0.577551	1.262252
H	-4.747323	2.133161	4.535547
H	-6.191778	0.445318	3.409463

Ag-Int4-β

C	-2.132693	1.871544	1.028724
C	-1.827809	2.521664	-0.123580
Ag	0.298840	-0.706284	1.104408
C	0.636714	-1.754868	-0.652875
N	1.824362	-2.273486	-1.079101
N	-0.269842	-2.222759	-1.551682
C	1.658944	-3.051665	-2.219899
H	2.487549	-3.545230	-2.701774
C	0.335503	-3.022032	-2.517093
H	-0.230299	-3.482791	-3.311071
C	1.840732	3.400151	0.189041
C	1.490551	3.924194	-1.255022
B	-0.361836	3.003274	-0.281549
O	0.522396	3.179156	0.758941
O	0.201908	3.296014	-1.508658
C	2.560034	2.048718	0.182580
H	3.584589	2.135516	-0.190119
H	2.597009	1.654581	1.201245
H	2.023484	1.324813	-0.433052
C	2.589451	4.391119	1.073083
H	2.758734	3.945371	2.057694
H	3.564119	4.638366	0.639286
H	2.024852	5.314366	1.215372
C	2.462925	3.501599	-2.350448
H	2.139442	3.910752	-3.312529
H	3.469595	3.878859	-2.142367
H	2.509790	2.416021	-2.445741
C	1.254064	5.437390	-1.306393
H	2.186204	5.998121	-1.189583
H	0.814065	5.696045	-2.273855
H	0.557327	5.751547	-0.524004
O	-0.060449	0.076305	2.961275
C	0.816631	1.007678	3.525371
H	0.841848	1.968488	2.979792
H	0.513419	1.243905	4.563998
H	1.866037	0.650992	3.591205
H	-1.320855	1.742648	1.741978
C	3.089988	-1.985712	-0.455992
C	3.921994	-1.027748	-1.056947
C	3.437013	-2.640589	0.735877
C	5.122242	-0.710611	-0.414635
C	4.647840	-2.286282	1.340006
C	5.497688	-1.322257	0.786702
H	5.774575	0.036980	-0.859328
H	4.930093	-2.774556	2.269581
C	-1.677579	-1.914508	-1.541295
C	-2.140870	-0.864034	-2.347451
C	-2.535531	-2.685984	-0.741617
C	-3.519030	-0.626919	-2.370668
C	-3.900865	-2.392606	-0.783319
C	-4.411341	-1.375510	-1.597158
H	-3.901414	0.172255	-2.999929
H	-4.580330	-2.965852	-0.158041
C	3.529516	-0.356277	-2.349709
C	6.805732	-0.976182	1.455492
C	2.534603	-3.671600	1.366091
C	-1.191207	0.018002	-3.119652
C	-5.894762	-1.105846	-1.647649
C	-2.001158	-3.770974	0.159028
H	2.499923	0.012086	-2.314093
H	4.189090	0.486974	-2.567058
H	3.589284	-1.050090	-3.196561
H	6.716226	-0.999445	2.546066
H	7.590523	-1.691876	1.179120
H	7.154502	0.019486	1.165115
H	1.725272	-3.191270	1.928671
H	2.066628	-4.315284	0.614984
H	3.094358	-4.304298	2.060360
H	-0.698822	0.733825	-2.450622
H	-0.405016	-0.556004	-3.619801
H	-1.724727	0.594128	-3.879998
H	-6.357040	-1.257569	-0.668434
H	-6.104506	-0.079803	-1.963591
H	-6.392999	-1.778593	-2.357702
H	-2.813295	-4.393497	0.543261
H	-1.288098	-4.419662	-0.360526
H	-1.475562	-3.336385	1.017650
C	-2.772356	2.744202	-1.283704
H	-3.364512	3.659977	-1.146719
H	-2.214802	2.871770	-2.216311
H	-3.474905	1.917081	-1.412612
C	-3.370765	1.202501	1.455566
C	-4.661116	1.508163	0.979105
C	-3.241826	0.181245	2.423499
C	-5.774016	0.794742	1.425424
H	-4.803688	2.324535	0.281804
C	-4.354690	-0.539544	2.851654
H	-2.251004	-0.022402	2.828501
C	-5.625723	-0.241322	2.351096
H	-6.761608	1.053634	1.052501
H	-4.232099	-1.328534	3.589380
H	-6.494691	-0.798152	2.692055

Ag-Int3-α

C	0.854665	-1.919424	-0.545107
C	2.148752	-1.768981	-0.127764
Ag	-0.658155	-0.473560	-0.220588
C	-2.365944	0.786826	-0.150912
N	-2.521528	2.108200	-0.440486
N	-3.641017	0.329596	-0.010761
C	-3.866219	2.466513	-0.481422
H	-4.182142	3.473756	-0.701072

C	-4.574553	1.341118	-0.209703
H	-5.635863	1.162195	-0.141347
C	2.450127	1.461893	1.834638
C	3.633962	0.603287	2.413081
B	2.513908	-0.585325	0.808944
O	2.094367	0.721548	0.635738
O	3.315974	-0.725466	1.924554
C	1.205664	1.468611	2.728986
H	1.363999	2.046671	3.644570
H	0.380021	1.918940	2.172060
H	0.913626	0.449996	3.000533
C	2.827241	2.885075	1.440046
H	1.949645	3.399988	1.043671
H	3.189933	3.443824	2.309547
H	3.602520	2.894659	0.671663
C	3.715200	0.559902	3.934672
H	4.556502	-0.068761	4.241703
H	3.874787	1.562898	4.344586
H	2.806810	0.142743	4.373567
C	4.996014	0.970969	1.813811
H	5.355396	1.939780	2.173821
H	5.723668	0.204824	2.095809
H	4.946223	0.998881	0.721351
O	1.572334	-0.104163	-2.958882
C	2.578660	0.855261	-2.657250
H	3.535537	0.379935	-2.391321
H	2.740611	1.456913	-3.558219
H	2.285540	1.519277	-1.835079
H	1.380299	-0.577033	-2.128446
C	-1.435204	3.024625	-0.670904
C	-1.095133	3.921187	0.357563
C	-0.759467	3.003163	-1.899717
C	-0.047933	4.815157	0.125937
C	0.292492	3.912160	-2.075417
C	0.664498	4.818451	-1.080983
H	0.230115	5.513716	0.911349
H	0.833728	3.902864	-3.018209
C	-3.964088	-1.041091	0.293381
C	-4.129762	-1.410908	1.637226
C	-4.073978	-1.961875	-0.759021
C	-4.425171	-2.748469	1.912616
C	-4.368322	-3.289766	-0.431545
C	-4.544676	-3.701450	0.893454
H	-4.558968	-3.054723	2.947391
H	-4.456556	-4.019330	-1.232760
C	-1.828691	3.912208	1.676485
C	1.838860	5.745452	-1.274508
C	-1.102623	2.035165	-3.003167
C	-3.956404	-0.403791	2.746504
C	-4.826830	-5.146424	1.224859
C	-3.843109	-1.548133	-2.190226
H	-1.895748	2.900484	2.089398
H	-1.319347	4.544103	2.408476
H	-2.855671	4.281748	1.574019
H	2.755974	5.291605	-0.877779
H	2.014283	5.961960	-2.332671
H	1.694606	6.695772	-0.750602
H	-0.440792	1.161254	-2.963434
H	-2.135758	1.685273	-2.943475
H	-0.957780	2.506333	-3.980691
H	-2.908872	-0.089023	2.821078
H	-4.550783	0.500150	2.576981
H	-4.250067	-0.828305	3.709811
H	-3.906391	-5.664441	1.521399
H	-5.531560	-5.237128	2.057920
H	-5.242526	-5.682871	0.366788
H	-4.370026	-0.621307	-2.439131
H	-2.775934	-1.371290	-2.371418
H	-4.176846	-2.326922	-2.880810
C	0.421069	-3.146302	-1.331429
H	-0.483760	-3.576570	-0.883431
H	0.136540	-2.863780	-2.355173
H	1.176773	-3.939313	-1.402956
C	3.267142	-2.694380	-0.500228
C	3.608202	-2.868002	-1.854735
C	4.041988	-3.358673	0.466627
C	4.668741	-3.693786	-2.230464
H	3.037178	-2.331579	-2.608521
C	5.100473	-4.187252	0.091307
H	3.810169	-3.212816	1.516735
C	5.418022	-4.361289	-1.258517
H	4.913514	-3.810770	-3.283352
H	5.681125	-4.698022	0.855738
H	6.244461	-5.004661	-1.548741

Ag-TS2-α

C	-1.459269	0.803384	2.046016
C	-1.868389	1.580100	0.984539
Ag	0.241975	-0.383041	0.838886
C	0.934844	-1.931630	-0.398533
N	2.202420	-2.313617	-0.714342
N	0.148602	-2.916165	-0.909379
C	2.205692	-3.520277	-1.409867
H	3.118843	-3.987156	-1.742605
C	0.907390	-3.901340	-1.532539
H	0.457954	-4.766835	-1.993006
C	1.210445	3.070799	-0.641111
C	0.085273	4.057073	-1.122569
B	-0.819346	2.438270	0.221309
O	0.548957	2.378343	0.450194
O	-1.125900	3.325450	-0.788835
C	1.573052	2.009703	-1.686275
H	2.119840	2.440072	-2.530695

H	2.206703	1.255135	-1.215128
H	0.675559	1.512343	-2.065760
C	2.475105	3.730641	-0.102536
H	3.178971	2.959025	0.223132
H	2.961238	4.330143	-0.879602
H	2.261358	4.374144	0.752443
C	0.077054	4.349132	-2.618830
H	-0.746630	5.028449	-2.858151
H	1.012159	4.828501	-2.926890
H	-0.059386	3.438092	-3.204732
C	0.050353	5.361721	-0.320402
H	0.900389	6.007009	-0.561451
H	-0.871264	5.901212	-0.556314
H	0.056697	5.159611	0.754470
O	0.874746	0.329407	3.133582
C	1.850119	1.326965	3.305710
H	1.918647	1.665395	4.355628
H	2.851289	0.955277	3.026860
H	1.650967	2.213203	2.681858
H	-0.160804	0.730369	2.692282
C	3.364731	-1.514321	-0.419585
C	3.973281	-0.815068	-1.479018
C	3.824450	-1.414252	0.902883
C	5.064522	0.003501	-1.182889
C	4.921245	-0.575334	1.144657
C	5.547980	0.143038	0.124559
H	5.540318	0.556144	-1.989482
H	5.288622	-0.484932	2.163819
C	-1.288192	-2.879144	-0.795476
C	-2.025502	-2.234918	-1.802425
C	-1.888825	-3.393178	0.361930
C	-3.404644	-2.114288	-1.622782
C	-3.274598	-3.247884	0.494813
C	-4.044679	-2.602274	-0.476769
H	-3.991691	-1.601897	-2.380316
H	-3.756950	-3.628711	1.391382
C	3.448207	-0.914970	-2.890541
C	6.694703	1.078908	0.419928
C	3.168343	-2.135494	2.053821
C	-1.343229	-1.624816	-3.001229
C	-5.524276	-2.382824	-0.284899
C	-1.065861	-4.015535	1.461411
H	2.366237	-0.755423	-2.926504
H	3.921845	-0.168173	-3.532754
H	3.639553	-1.900700	-3.329650
H	6.344452	2.117390	0.473908
H	7.171713	0.844836	1.376036
H	7.459732	1.037192	-0.362475
H	2.393800	-1.505459	2.510787
H	2.695069	-3.068991	1.738556
H	3.906926	-2.370049	2.826349
H	-0.786541	-0.725590	-2.710921
H	-0.624887	-2.310727	-3.461673
H	-2.073595	-1.334835	-3.760971
H	-5.725344	-1.328301	-0.061532
H	-6.088181	-2.633462	-1.190232
H	-5.919699	-2.982924	0.539719
H	-0.315987	-4.711182	1.071685
H	-0.527937	-3.241735	2.022752
H	-1.701509	-4.557445	2.166452
C	-2.426000	-0.059925	2.832625
H	-1.921049	-0.965155	3.189572
H	-2.752158	0.471561	3.738347
H	-3.330326	-0.358296	2.286126
C	-3.279780	1.640811	0.486453
C	-4.369838	1.889312	1.338213
C	-3.552968	1.432332	-0.878270
C	-5.679329	1.905592	0.853383
H	-4.184179	2.079523	2.390751
C	-4.858922	1.440665	-1.366095
H	-2.723858	1.256495	-1.556925
C	-5.932105	1.674961	-0.500777
H	-6.503125	2.101777	1.535009
H	-5.040989	1.267367	-2.423925
H	-6.950744	1.685191	-0.879058

Ag-Int4-α

C	-2.192526	0.550620	1.906102
C	-2.591218	1.331185	0.867703
Ag	1.011739	-0.793067	1.298404
C	1.304593	-1.673508	-0.554767
N	2.459086	-1.709554	-1.277806
N	0.444929	-2.454378	-1.262506
C	2.323184	-2.502654	-2.412615
H	3.134460	-2.651212	-3.107028
C	1.050741	-2.975182	-2.401505
H	0.521341	-3.620301	-3.084427
C	0.389096	3.616539	0.406842
C	-0.444479	3.925343	-0.890245
B	-1.532116	2.371564	0.393178
O	-0.583706	2.915577	1.229578
O	-1.401233	2.826570	-0.905287
C	1.549192	2.648543	0.156951
H	2.349883	3.116882	-0.422664
H	1.960340	2.323668	1.115299
H	1.203885	1.760165	-0.375791
C	0.876964	4.841545	1.172135
H	1.429970	4.521197	2.059945
H	1.548905	5.444259	0.552046
H	0.047919	5.469967	1.502889
C	0.346110	3.896400	-2.192895
H	-0.319770	4.107653	-3.035244
H	1.133381	4.657262	-2.183880

H	0.805102	2.920905	-2.362524
C	-1.264484	5.215529	-0.789128
H	-0.628923	6.104623	-0.839570
H	-1.975855	5.250814	-1.619142
H	-1.834818	5.246064	0.143628
O	0.770372	-0.138305	3.218013
C	1.289680	1.087443	3.638770
H	1.187290	1.193976	4.736711
H	2.371841	1.219391	3.424664
H	0.770722	1.956725	3.193980
H	-1.229141	0.772145	2.361751
C	3.635041	-0.952090	-0.937108
C	3.859417	0.262381	-1.607380
C	4.498668	-1.431155	0.058212
C	4.983259	1.008719	-1.245782
C	5.611656	-0.646492	0.382844
C	5.865944	0.574465	-0.249036
H	5.171765	1.953104	-1.751009
H	6.293712	-1.000630	1.151857
C	-0.934027	-2.665952	-0.900830
C	-1.912852	-1.835113	-1.468496
C	-1.243583	-3.674554	0.023292
C	-3.241478	-2.046657	-1.089463
C	-2.586736	-3.841543	0.375551
C	-3.595082	-3.039181	-0.169235
H	-4.012764	-1.400964	-1.498023
H	-2.847230	-4.609092	1.100257
C	2.916621	0.744324	-2.681705
C	7.049402	1.422523	0.148804
C	4.234953	-2.729775	0.778200
C	-1.550120	-0.707686	-2.403207
C	-5.040301	-3.224993	0.224327
C	-0.160282	-4.516882	0.649165
H	1.875981	0.701955	-2.347151
H	3.144133	1.774244	-2.966393
H	2.986427	0.126664	-3.584797
H	6.750313	2.194350	0.868921
H	7.834480	0.823661	0.619862
H	7.484257	1.936598	-0.714475
H	3.484337	-2.591751	1.565652
H	3.854103	-3.501711	0.102454
H	5.147292	-3.104818	1.249705
H	-1.231535	0.175589	-1.837138
H	-0.734951	-0.975302	-3.081775
H	-2.412307	-0.413484	-3.005721
H	-5.548012	-2.259604	0.315927
H	-5.584276	-3.807091	-0.530565
H	-5.133430	-3.754196	1.177389
H	0.529113	-4.919046	-0.100903
H	0.439909	-3.921399	1.347555
H	-0.588289	-5.355794	1.203821
C	-2.847772	-0.661649	2.495694
H	-2.078505	-1.418172	2.686968
H	-3.296785	-0.428868	3.471780
H	-3.619167	-1.094102	1.856636
C	-3.902897	1.187291	0.178965
C	-5.073316	0.868006	0.896617
C	-4.036629	1.400817	-1.207737
C	-6.302273	0.708439	0.254775
H	-5.024545	0.767594	1.975364
C	-5.264181	1.240989	-1.850986
H	-3.168154	1.694603	-1.782703
C	-6.403792	0.881369	-1.127482
H	-7.184415	0.461880	0.840081
H	-5.330287	1.403054	-2.924045
H	-7.359401	0.757232	-1.629361

Ag-Int5

Ag	-0.835318	0.069336	-0.573975
C	-1.630619	-1.680752	0.213884
N	-2.963690	-1.909870	0.369274
N	-1.034159	-2.733119	0.835664
C	-3.199158	-3.082580	1.077669
H	-4.196573	-3.425169	1.302546
C	-1.981315	-3.602167	1.372172
H	-1.695630	-4.491542	1.910512
C	3.741264	-0.147951	-2.004093
C	4.557975	0.517489	-0.838343
C	0.142284	3.064733	1.659290
C	1.103812	4.144501	1.043228
O	3.514823	1.141873	-0.049096
O	2.569815	0.704534	-2.080706
O	0.567810	1.878057	0.977360
O	1.215401	3.731673	-0.316703
C	5.485437	1.641079	-1.316078
H	5.858267	2.187304	-0.444795
H	6.343547	1.254537	-1.875168
H	4.943962	2.350513	-1.948193
C	5.318108	-0.457883	0.054703
H	6.059891	-1.025781	-0.517680
H	5.846881	0.093278	0.838826
H	4.634425	-1.155263	0.539717
C	3.234023	-1.551964	-1.658719
H	2.508624	-1.864477	-2.416235
H	4.043897	-2.287910	-1.635928
H	2.730122	-1.556062	-0.690571
C	4.435455	-0.153268	-3.362655
H	5.371185	-0.721823	-3.323396
H	3.785921	-0.622913	-4.108296
H	4.658074	0.859264	-3.704634
C	2.506332	4.093762	1.676548
H	3.181976	4.708888	1.073511
H	2.519328	4.475653	2.703722
H	2.890918	3.070713	1.666323

C	0.564225	5.574526	1.086116
H	0.372569	5.899350	2.115531
H	1.296851	6.260811	0.648011
H	-0.360044	5.665767	0.511326
C	0.300717	2.840771	3.162955
H	0.116197	3.763686	3.724862
H	-0.418783	2.089226	3.507948
H	1.302269	2.478419	3.404179
C	-1.333006	3.347231	1.326874
H	-1.929395	2.463864	1.575913
H	-1.728682	4.195599	1.896868
H	-1.451525	3.539843	0.259094
B	1.029662	2.281576	-0.372211
B	2.408002	1.354315	-0.859392
O	-0.183761	1.975169	-1.304203
C	-0.077432	2.385119	-2.660244
H	0.132822	3.461124	-2.700168
H	0.722064	1.848099	-3.186138
H	-1.030531	2.195897	-3.168171
C	0.386174	-2.935591	0.981036
C	0.992370	-3.948531	0.213853
C	1.101848	-2.168506	1.912238
C	2.345640	-4.207953	0.428210
C	2.463989	-2.462787	2.077629
C	3.095939	-3.482184	1.363987
H	2.830389	-4.987815	-0.154415
H	3.036198	-1.877859	2.792812
C	-3.984058	-0.989386	-0.063005
C	-4.527316	-1.121889	-1.347227
C	-4.370977	0.032941	0.820367
C	-5.499030	-0.193103	-1.738692
C	-5.340614	0.936379	0.381948
C	-5.916017	0.838815	-0.892336
H	-5.934381	-0.277769	-2.731301
H	-5.651480	1.737889	1.047780
C	0.209868	-4.718009	-0.822498
H	-0.583196	-5.325978	-0.373161
H	-0.273358	-4.040593	-1.535288
H	0.865932	-5.387265	-1.384440
C	0.469775	-1.052604	2.705853
H	0.540591	-0.101511	2.162816
H	-0.587852	-1.241949	2.911208
H	0.985171	-0.924468	3.662367
C	4.558091	-3.795934	1.567091
H	4.697945	-4.834445	1.889741
H	5.120249	-3.671186	0.634763
H	5.011784	-3.145743	2.319832
C	-4.056250	-2.201553	-2.288779
H	-3.057367	-1.969491	-2.676864
H	-3.987927	-3.174907	-1.792760
H	-4.732388	-2.299687	-3.141918
C	-3.740375	0.153845	2.185217
H	-3.901823	-0.749254	2.784976
H	-2.657115	0.294491	2.106076
H	-4.153562	1.003778	2.733146
C	-6.976570	1.821837	-1.324090
H	-7.913686	1.653118	-0.779180
H	-6.668675	2.853050	-1.120670
H	-7.193744	1.738342	-2.392634

Ag-TS3

Ag	-0.279157	-0.273857	-0.410117
C	-0.507490	-2.246446	0.278542
N	-1.704732	-2.849468	0.511988
N	0.409674	-3.109998	0.789952
C	-1.539119	-4.068876	1.159206
H	-2.378031	-4.688871	1.432349
C	-0.203177	-4.234472	1.335405
H	0.363893	-5.028260	1.794828
C	3.117137	1.450790	-1.770920
C	3.356386	2.165739	-0.396512
C	-1.696783	3.251252	1.443429
C	-0.857196	4.331240	0.666925
O	2.106149	1.903333	0.302875
O	1.671434	1.549358	-1.913149
O	-1.060585	2.036000	1.031976
O	-0.547762	3.670361	-0.567749
C	3.481843	3.687948	-0.526932
H	3.453345	4.135782	0.469942
H	4.422202	3.975860	-1.007348
H	2.648644	4.100347	-1.101966
C	4.506347	1.605188	0.432132
H	5.459046	1.720134	-0.096445
H	4.578214	2.149583	1.379029
H	4.353976	0.549951	0.662083
C	3.458968	-0.043291	-1.735949
H	3.059979	-0.521697	-2.635451
H	4.540260	-0.211007	-1.708769
H	3.009758	-0.529314	-0.867558
C	3.770588	2.119715	-2.975233
H	4.859867	2.148069	-2.862729
H	3.539456	1.553021	-3.882759
H	3.406437	3.139165	-3.115035
C	0.460190	4.670788	1.380692
H	1.077355	5.274777	0.708680
H	0.296013	5.244062	2.299833
H	1.011632	3.758105	1.614231
C	-1.620178	5.616858	0.345195
H	-1.966433	6.113072	1.259367
H	-0.964894	6.313109	-0.188862
H	-2.482978	5.416951	-0.293665
C	-1.615030	3.346050	2.966268
H	-1.979452	4.314802	3.326765

H	-2.234465	2.564559	3.420118
H	-0.589985	3.204423	3.315213
C	-3.170428	3.222235	1.002686
H	-3.639127	2.319638	1.407656
H	-3.727861	4.092516	1.366638
H	-3.244846	3.170895	-0.084976
B	-0.635242	2.224226	-0.355799
B	1.113478	1.719928	-0.648317
O	-1.624238	1.583988	-1.277338
C	-1.543573	1.929518	-2.648633
H	-1.728579	3.002061	-2.796000
H	-0.558964	1.694194	-3.079712
H	-2.307838	1.364976	-3.197210
C	1.830767	-2.875526	0.792026
C	2.614576	-3.479499	-0.203438
C	2.381679	-2.057541	1.790852
C	3.992615	-3.245153	-0.179448
C	3.765664	-1.855517	1.771025
C	4.584468	-2.434562	0.795497
H	4.615288	-3.699739	-0.945838
H	4.211031	-1.223933	2.535245
C	-2.975019	-2.287673	0.121887
C	-3.572287	-2.757508	-1.059823
C	-3.553654	-1.279027	0.908039
C	-4.789442	-2.191364	-1.445752
C	-4.774041	-0.746666	0.476572
C	-5.401468	-1.181233	-0.693734
H	-5.266129	-2.540836	-2.358330
H	-5.237835	0.038171	1.068831
C	1.988764	-4.334864	-1.276406
H	1.433785	-5.179041	-0.852842
H	1.277801	-3.756308	-1.876654
H	2.750695	-4.735608	-1.949498
C	1.518917	-1.394426	2.834182
H	0.964866	-0.552760	2.403724
H	0.782468	-2.087031	3.254352
H	2.129772	-1.005429	3.652857
C	6.064399	-2.144062	0.765642
H	6.623146	-2.940667	0.265162
H	6.262640	-1.211883	0.221557
H	6.472084	-2.021680	1.774058
C	-2.906997	-3.818389	-1.901287
H	-1.938607	-3.470559	-2.278461
H	-2.716571	-4.734786	-1.332255
H	-3.527820	-4.079722	-2.761870
C	-2.884948	-0.732584	2.144412
H	-2.286282	-1.488137	2.660743
H	-2.222973	0.102447	1.880104
H	-3.631839	-0.352412	2.847850
C	-6.691844	-0.548234	-1.153809
H	-7.315094	-0.245320	-0.306480
H	-6.490126	0.352939	-1.746112
H	-7.276104	-1.228578	-1.781246

Cu-Catalyst

C	0.393236	1.593378	0.091236
C	1.527196	3.508505	0.614096
C	0.194145	3.811064	0.611599
N	1.625197	2.155781	0.293080
N	-0.476967	2.632897	0.287684
B	-0.532613	-2.242506	-0.211214
O	0.181293	-3.257971	-0.857479
O	-1.654916	-2.795795	0.418007
C	-0.355119	-4.544976	-0.453381
C	-1.811431	-4.172047	-0.015549
C	-2.373571	-4.993014	1.141825
H	-3.385515	-4.650842	1.381309
H	-2.429948	-6.055696	0.880311
H	-1.765088	-4.887551	2.042113
C	-2.807027	-4.160148	-1.183640
H	-3.040678	-5.170215	-1.536267
H	-3.736540	-3.687558	-0.852319
H	-2.414775	-3.577077	-2.021418
C	-0.257014	-5.512481	-1.629513
H	0.794752	-5.706119	-1.863827
H	-0.731651	-6.471082	-1.391195
H	-0.727342	-5.103909	-2.526061
C	0.513363	-5.042552	0.710740
H	0.223595	-6.045248	1.042089
H	1.557339	-5.075315	0.385165
H	0.449168	-4.358746	1.561800
H	-0.330395	4.732157	0.810297
H	2.396546	4.113573	0.817243
C	-1.909211	2.520775	0.169053
C	-2.637535	1.920399	1.209146
C	-2.529480	3.016798	-0.988930
C	-4.025399	1.828249	1.062930
C	-3.920228	2.907903	-1.083074
C	-4.683167	2.313954	-0.071817
H	-4.605086	1.363166	1.856658
H	-4.416910	3.286931	-1.973027
C	2.870821	1.440799	0.180994
C	3.655328	1.631925	-0.968246
C	3.266134	0.586590	1.222575
C	4.872339	0.949273	-1.049986
C	4.489805	-0.078498	1.089760
C	5.305108	0.089958	-0.033688
H	5.492035	1.085055	-1.933207
H	4.811670	-0.744971	1.886314
C	-1.952127	1.362485	2.430275
C	-6.178500	2.168074	-0.215302
C	-1.722772	3.629172	-2.107122
C	3.198697	2.538872	-2.084495

C	6.604716	-0.666395	-0.166388
C	2.393314	0.361968	2.430735
H	-2.674997	1.156115	3.223872
H	-1.442084	0.422809	2.184769
H	-1.194880	2.048346	2.823471
H	-6.684673	2.245866	0.752244
H	-6.596590	2.929996	-0.880305
H	-6.435628	1.188517	-0.637503
H	-0.952132	2.936643	-2.462211
H	-2.363893	3.883024	-2.955084
H	-1.207812	4.542742	-1.789885
H	1.551824	-0.294774	2.175594
H	2.958262	-0.109611	3.239234
H	1.967806	1.297262	2.807713
H	7.351895	-0.089418	-0.720859
H	7.025830	-0.917760	0.811897
H	6.453828	-1.608501	-0.708357
H	3.823745	2.407633	-2.971383
H	2.162171	2.329250	-2.366656
H	3.244932	3.595867	-1.796541
Cu	-0.049795	-0.308580	-0.147327

Cu-Int1-β

C	-0.366920	-0.567705	-1.127337
C	-1.174274	-0.822726	-3.246230
C	-0.065229	-1.594803	-3.143058
N	-1.344905	-0.207110	-2.007549
N	0.417163	-1.421701	-1.847266
B	1.729640	1.149684	0.715264
O	2.134893	2.074312	-0.254324
O	2.691394	1.080771	1.721182
C	3.272813	2.824688	0.254374
C	3.864078	1.841984	1.323641
C	4.450884	2.510044	2.564280
H	4.815931	1.744794	3.256535
H	5.294878	3.157847	2.302471
H	3.703759	3.106540	3.091518
C	4.865865	0.843231	0.735161
H	5.813412	1.322036	0.466972
H	5.068676	0.064433	1.476168
H	4.450774	0.356012	-0.149044
C	4.201037	3.156542	-0.909909
H	3.688272	3.820808	-1.612672
H	5.103462	3.668278	-0.557934
H	4.500338	2.260256	-1.456040
C	2.709305	4.114284	0.862903
H	3.501535	4.780304	1.219601
H	2.132543	4.643758	0.098695
H	2.036357	3.889772	1.695039
H	0.423097	-2.243253	-3.852860
H	-1.852811	-0.652050	-4.066642
C	-1.457513	-0.336970	2.067766
C	-0.518848	0.373678	2.532300
C	1.618735	-2.023920	-1.338308
C	1.514547	-3.082663	-0.425587
C	2.859666	-1.515575	-1.757858
C	2.699582	-3.641236	0.067152
C	4.013483	-2.119868	-1.251934
C	3.954813	-3.177270	-0.336281
H	2.636359	-4.458847	0.781027
H	4.983881	-1.742925	-1.566463
C	-2.425213	0.687449	-1.690236
C	-2.178395	2.067851	-1.663764
C	-3.689348	0.143576	-1.409275
C	-3.248091	2.912582	-1.342315
C	-4.725935	1.029708	-1.107276
C	-4.524684	2.414603	-1.065898
H	-3.075490	3.985655	-1.313220
H	-5.709426	0.626754	-0.878854
C	2.946204	-0.312760	-2.664107
C	0.167389	-3.576044	0.037148
C	5.225644	-3.772281	0.220191
C	-0.808233	2.623555	-1.956422
C	-3.909552	-1.347896	-1.390808
C	-5.670760	3.339567	-0.732757
H	0.269971	-4.462289	0.668855
H	-0.344869	-2.798252	0.611563
H	-0.486140	-3.827651	-0.805439
H	5.729273	-3.061952	0.887194
H	5.029041	-4.682417	0.794126
H	5.934566	-4.021450	-0.577441
H	2.367771	-0.440726	-3.584426
H	2.553886	0.571774	-2.149353
H	3.983643	-0.113001	-2.946266
H	-0.097299	2.384708	-1.158856
H	-0.393144	2.203367	-2.879340
H	-0.845318	3.711162	-2.064843
H	-4.938350	-1.586431	-1.113812
H	-3.703078	-1.803921	-2.365389
H	-3.249318	-1.826692	-0.661160
H	-6.436493	3.321019	-1.518042
H	-6.162054	3.041331	0.200396
H	-5.334867	4.374418	-0.619950
C	0.125993	1.087829	3.658553
H	1.186796	0.833624	3.716160
H	0.069963	2.170561	3.498701
H	-0.366078	0.851321	4.610466
C	-2.720171	-1.026461	2.092529
C	-2.791778	-2.431652	2.205417
C	-3.927171	-0.303340	1.984513
C	-4.023784	-3.082816	2.212188
H	-1.872959	-3.002478	2.294515
C	-5.154007	-0.962740	1.993310

H	-3.884724	0.775390	1.875627
C	-5.212808	-2.355318	2.104815
H	-4.055254	-4.165537	2.303535
H	-6.070897	-0.384829	1.909734
H	-6.171418	-2.866230	2.109148
Cu	-0.012981	0.116718	0.674526

Cu-TS1-β

C	0.055738	-0.435827	-1.577470
C	-0.390876	-0.415958	-3.810839
C	0.748597	-1.129961	-3.632091
N	-0.798575	0.000105	-2.546460
N	1.010162	-1.130863	-2.262495
B	0.917979	1.175546	1.260591
O	0.743647	2.542256	1.110518
O	2.142938	0.878558	1.831774
C	1.890819	3.225508	1.698850
C	2.966109	2.077344	1.767247
C	3.872216	2.112617	2.993162
H	4.576377	1.275862	2.955713
H	4.451564	3.041673	3.023686
H	3.300680	2.028339	3.919459
C	3.804142	1.959556	0.489248
H	4.517298	2.783958	0.390199
H	4.357755	1.017371	0.516236
H	3.161539	1.941894	-0.393629
C	2.258133	4.399831	0.795960
H	1.444527	5.131674	0.795823
H	3.163529	4.900167	1.155715
H	2.422185	4.081531	-0.234983
C	1.459760	3.744347	3.073982
H	2.242636	4.354689	3.534946
H	0.566728	4.364571	2.954327
H	1.213533	2.926387	3.752842
H	1.390453	-1.635335	-4.335559
H	-0.947267	-0.168958	-4.700953
C	-1.473945	-0.858386	1.616875
C	-0.695353	0.059925	2.136405
C	2.162301	-1.721912	-1.639947
C	2.001884	-2.879977	-0.860422
C	3.407669	-1.096739	-1.804773
C	3.137640	-3.403039	-0.235762
C	4.514130	-1.661471	-1.161062
C	4.397803	-2.808082	-0.369229
H	3.032704	-4.296339	0.375663
H	5.485009	-1.184386	-1.272473
C	-1.973107	0.774648	-2.248497
C	-1.847308	2.161921	-2.096704
C	-3.187651	0.100232	-2.039110
C	-2.998716	2.886183	-1.763194
C	-4.306786	0.866106	-1.706489
C	-4.231908	2.258745	-1.566524
H	-2.922290	3.963863	-1.641216
H	-5.256225	0.363434	-1.537425
C	3.553478	0.151708	-2.639752
C	0.653160	-3.531961	-0.686649
C	5.602355	-3.410565	0.312625
C	-0.506471	2.842008	-2.215132
C	-3.253870	-1.404070	-2.094935
C	-5.466266	3.053120	-1.214322
H	0.757463	-4.531857	-0.257011
H	0.012664	-2.939951	-0.022247
H	0.121814	-3.620667	-1.640048
H	6.445461	-2.713835	0.332073
H	5.374152	-3.693585	1.345698
H	5.934980	-4.319129	-0.204874
H	3.518736	-0.072991	-3.712510
H	2.748584	0.864039	-2.437760
H	4.506626	0.646851	-2.437823
H	0.080600	2.670645	-1.304590
H	0.073024	2.460112	-3.061587
H	-0.625667	3.921940	-2.339993
H	-4.285837	-1.755922	-2.021371
H	-2.819739	-1.801490	-3.018383
H	-2.689620	-1.830241	-1.257666
H	-6.193770	3.039710	-2.035371
H	-5.968105	2.635045	-0.334688
H	-5.226102	4.098141	-0.998996
C	-0.624774	0.735111	3.476609
H	0.364506	0.624517	3.933347
H	-0.836630	1.806454	3.397540
H	-1.368359	0.283971	4.144257
C	-2.579242	-1.671197	2.074781
C	-2.399827	-2.706740	3.018312
C	-3.877048	-1.474528	1.550737
C	-3.470880	-3.506679	3.415945
H	-1.407820	-2.877761	3.428107
C	-4.944732	-2.266011	1.966226
H	-4.026719	-0.690956	0.813101
C	-4.751401	-3.291937	2.898175
H	-3.305622	-4.299117	4.142305
H	-5.935541	-2.086820	1.554688
H	-5.583891	-3.915430	3.212070
Cu	-0.209111	-0.156009	0.269356

Cu-Int2-β

C	-0.726642	-1.699665	0.169301
C	-1.836127	-3.683789	0.331709
C	-0.586868	-3.877389	0.825977
N	-1.900819	-2.351776	-0.064428
N	0.074963	-2.657185	0.718957
B	1.935088	2.260341	-0.764556

O	1.931341	1.059895	-1.461569
O	3.181309	2.864262	-0.800138
C	3.173190	0.986964	-2.208570
C	4.117332	1.903090	-1.353108
C	5.184552	2.653356	-2.141492
H	5.780950	3.271040	-1.463019
H	5.860565	1.954203	-2.645175
H	4.740777	3.311796	-2.890482
C	4.745014	1.159003	-0.166854
H	5.521044	0.458884	-0.491853
H	5.199367	1.889280	0.509337
H	3.985650	0.603289	0.389976
C	3.603968	-0.470670	-2.313688
H	2.875117	-1.027443	-2.910445
H	4.578013	-0.552856	-2.808147
H	3.665336	-0.939584	-1.332075
C	2.886121	1.558804	-3.602682
H	3.757614	1.484986	-4.260672
H	2.064278	0.995331	-4.054015
H	2.581525	2.607782	-3.542562
H	-0.113161	-4.751530	1.243325
H	-2.674166	-4.354454	0.227509
C	-0.353061	2.104067	0.265052
C	0.715011	2.892842	-0.051072
C	1.422494	-2.409785	1.160636
C	1.629579	-1.696010	2.353281
C	2.485054	-2.867678	0.368847
C	2.949465	-1.443401	2.738138
C	3.787911	-2.598858	0.803246
C	4.039410	-1.881643	1.976952
H	3.129520	-0.884653	3.653539
H	4.623230	-2.942139	0.198072
C	-3.067006	-1.706191	-0.610334
C	-3.318305	-1.811759	-1.985285
C	-3.891476	-0.962652	0.250807
C	-4.446945	-1.158770	-2.493814
C	-5.002716	-0.325399	-0.305910
C	-5.297965	-0.413513	-1.671826
H	-4.656679	-1.226175	-3.558417
H	-5.645183	0.265636	0.341600
C	2.232168	-3.597693	-0.926488
C	0.475060	-1.188578	3.179279
C	5.448898	-1.548364	2.400329
C	-2.375040	-2.560855	-2.893292
C	-3.554879	-0.807767	1.711460
C	-6.514813	0.283031	-2.231035
H	0.806602	-0.908764	4.182687
H	0.028818	-0.302962	2.710558
H	-0.319975	-1.934985	3.273070
H	6.188027	-2.117929	1.829429
H	5.657996	-0.483130	2.244806
H	5.607835	-1.755817	3.464137
H	1.746604	-4.566139	-0.761371
H	1.572637	-3.018043	-1.580673
H	3.167508	-3.779508	-1.461393
H	-1.409961	-2.044416	-2.956761
H	-2.171515	-3.574019	-2.531019
H	-2.782354	-2.637083	-3.904762
H	-4.365280	-0.313594	2.251798
H	-3.357592	-1.774101	2.188114
H	-2.653632	-0.195502	1.828376
H	-7.438822	-0.192506	-1.879735
H	-6.551821	1.330717	-1.912703
H	-6.525524	0.263272	-3.324548
C	0.816991	4.382419	0.251560
H	1.592830	4.585498	1.002568
H	1.112666	4.947230	-0.642397
H	-0.122247	4.804444	0.620213
C	-1.533403	2.625645	1.001306
C	-1.437627	3.113965	2.320555
C	-2.821196	2.548862	0.430874
C	-2.572648	3.502452	3.034877
H	-0.456308	3.175824	2.783968
C	-3.951994	2.961132	1.133833
H	-2.921308	2.138369	-0.570497
C	-3.837692	3.434422	2.445337
H	-2.467465	3.867011	4.054230
H	-4.929185	2.899905	0.660424
H	-4.720666	3.741251	2.999464
Cu	-0.443869	0.194882	0.033545

Cu-Int1-α

C	-1.051510	1.545281	0.065055
C	-1.206244	3.783698	-0.345018
C	-2.386864	3.226315	-0.708981
N	-0.403205	2.744437	0.122188
N	-2.275974	1.860449	-0.450050
B	1.084685	-0.689497	-0.849386
O	2.362394	-0.143910	-0.946815
O	0.903711	-1.635776	-1.868818
C	3.142757	-0.917727	-1.895648
C	2.029659	-1.574536	-2.785324
C	2.338791	-2.986900	-3.272719
H	1.493791	-3.371030	-3.852936
H	3.224449	-2.994526	-3.917769
H	2.510583	-3.670711	-2.439426
C	1.596228	-0.697288	-3.966293
H	2.367116	-0.644453	-4.741809
H	0.691109	-1.121251	-4.411320
H	1.362527	0.318432	-3.636905
C	4.088460	0.025711	-2.633223
H	4.799003	0.461821	-1.924327
H	4.660622	-0.510259	-3.398887

H	3.548903	0.844437	-3.112397
C	3.945773	-1.939047	-1.080349
H	4.604236	-2.543514	-1.712847
H	4.562526	-1.403753	-0.352201
H	3.280248	-2.604708	-0.524115
H	-3.282889	3.659154	-1.124019
H	-0.860993	4.804874	-0.371194
C	-1.171849	-0.892954	2.201567
C	-0.283647	-1.696961	1.778502
C	-3.321566	0.896701	-0.657431
C	-4.383621	0.849024	0.259822
C	-3.238472	0.019935	-1.751289
C	-5.372413	-0.121348	0.068262
C	-4.252943	-0.931754	-1.898783
C	-5.323150	-1.020509	-1.002437
H	-6.195929	-0.178347	0.776143
H	-4.200314	-1.624368	-2.735521
C	0.961230	2.893084	0.554559
C	1.957515	3.040306	-0.419808
C	1.257324	2.846234	1.926067
C	3.281257	3.167936	0.011037
C	2.595380	2.971724	2.307470
C	3.619635	3.133191	1.366111
H	4.066548	3.274343	-0.733111
H	2.845053	2.934440	3.365517
C	-2.086292	0.073751	-2.722149
C	-4.453225	1.806548	1.424501
C	-6.409583	-2.049367	-1.203252
C	1.613880	2.997700	-1.885435
C	0.175211	2.621208	2.951540
C	5.056338	3.239817	1.816724
H	-5.229887	1.504103	2.131829
H	-3.501781	1.852007	1.961426
H	-4.682626	2.826856	1.095368
H	-7.135123	-1.715474	-1.956025
H	-5.997860	-3.001938	-1.552192
H	-6.961745	-2.237501	-0.277615
H	-1.803370	1.104512	-2.958348
H	-1.201150	-0.417271	-2.304252
H	-2.343628	-0.433996	-3.656126
H	1.132980	2.044028	-2.121905
H	0.922367	3.796582	-2.174286
H	2.512488	3.090162	-2.499848
H	0.535912	2.848601	3.958356
H	-0.708834	3.236590	2.754734
H	-0.147902	1.574491	2.931045
H	5.189203	4.049954	2.543280
H	5.383418	2.314069	2.304806
H	5.730216	3.428222	0.975958
C	0.474717	-2.925149	1.941133
C	0.907632	-3.677673	0.835039
C	0.772636	-3.391432	3.236217
C	1.607916	-4.868694	1.023102
H	0.695883	-3.307704	-0.162264
C	1.484785	-4.576159	3.417376
H	0.448354	-2.810622	4.095089
C	1.902931	-5.322314	2.312099
H	1.929778	-5.443397	0.158356
H	1.713590	-4.917606	4.423602
H	2.457054	-6.246029	2.454520
C	-2.271535	-0.562188	3.135033
H	-2.160671	0.446094	3.549497
H	-3.232848	-0.587140	2.610047
H	-2.324307	-1.270584	3.972786
Cu	-0.312999	-0.200820	0.525443

Cu-TS1-α

C	-1.475680	1.390378	-0.058995
C	-2.175027	3.415985	-0.833111
C	-3.212530	2.552720	-0.965257
N	-1.121825	2.689688	-0.280535
N	-2.769423	1.320625	-0.487690
B	0.994501	-0.791564	-0.460667
O	2.177312	-0.133471	-0.727901
O	0.773383	-1.833248	-1.346264
C	2.908616	-0.864027	-1.752869
C	1.793431	-1.790655	-2.386264
C	2.236528	-3.223635	-2.670643
H	1.391045	-3.795284	-3.065913
H	3.038084	-3.245418	-3.416591
H	2.584310	-3.721555	-1.764693
C	1.129801	-1.194920	-3.632391
H	1.823899	-1.146439	-4.476862
H	0.282673	-1.823223	-3.921744
H	0.747890	-0.190061	-3.437041
C	3.520150	0.156368	-2.710108
H	4.226549	0.787095	-2.161846
H	4.066003	-0.340462	-3.519276
H	2.762970	0.808042	-3.147649
C	4.024248	-1.637136	-1.043528
H	4.664454	-2.161423	-1.760197
H	4.642181	-0.929530	-0.483399
H	3.624961	-2.362067	-0.334660
H	-4.211438	2.688596	-1.347870
H	-2.081035	4.462355	-1.076132
C	-0.070546	-0.718999	2.511509
C	0.704472	-1.279031	1.603404
C	-3.569563	0.129175	-0.406682
C	-4.472973	-0.001273	0.658576
C	-3.400245	-0.874243	-1.374190
C	-5.231931	-1.173901	0.730909
C	-4.177618	-2.029701	-1.254857
C	-5.098287	-2.196772	-0.213690

H	-5.935184	-1.293403	1.551473
H	-4.058816	-2.819270	-1.993340
C	0.191001	3.205781	-0.001490
C	1.111622	3.293895	-1.053835
C	0.520834	3.559212	1.316802
C	2.393958	3.770873	-0.764820
C	1.815751	4.024831	1.557560
C	2.763444	4.138204	0.532309
H	3.123036	3.838231	-1.568393
H	2.093596	4.301554	2.572088
C	-2.387079	-0.730751	-2.480791
C	-4.601505	1.079006	1.703470
C	-5.938873	-3.448296	-0.131879
C	0.737351	2.830184	-2.437711
C	-0.476313	3.399440	2.435766
C	4.157918	4.628953	0.838328
H	-5.297994	0.778899	2.490446
H	-3.632067	1.289657	2.167447
H	-4.963152	2.020142	1.273899
H	-6.713679	-3.452945	-0.908869
H	-5.330247	-4.346933	-0.279521
H	-6.440583	-3.535646	0.836285
H	-2.384679	0.279368	-2.902598
H	-1.380030	-0.930613	-2.101186
H	-2.590886	-1.439196	-3.288736
H	0.475476	1.767313	-2.414758
H	-0.131725	3.367069	-2.833187
H	1.565487	2.968182	-3.136967
H	-0.111610	3.866035	3.354673
H	-1.444770	3.845247	2.184272
H	-0.651563	2.335193	2.633924
H	4.136800	5.590147	1.364803
H	4.689409	3.920715	1.485126
H	4.750641	4.756588	-0.072051
C	1.732724	-2.334688	1.667025
C	1.563878	-3.578957	1.034241
C	2.898331	-2.119330	2.423749
C	2.530796	-4.576629	1.158858
H	0.678224	-3.742788	0.430507
C	3.866998	-3.117171	2.542715
H	3.038546	-1.157112	2.907159
C	3.689490	-4.348693	1.908090
H	2.382323	-5.533653	0.665142
H	4.764621	-2.928928	3.126025
H	4.445441	-5.124188	1.996023
C	-0.203247	-1.010203	3.972557
H	0.028137	-0.116499	4.566336
H	-1.238245	-1.275498	4.221606
H	0.450970	-1.824602	4.321895
Cu	-0.414445	0.042404	0.724207

C	1.824546	1.322159	-0.122700
C	2.793345	3.380307	0.022207
C	3.750959	2.464201	0.309887
N	1.624579	2.667229	-0.235132
N	3.142810	1.217257	0.216178
B	-2.205867	-1.192085	0.285594
O	-3.029707	-0.279397	-0.345433
O	-2.235753	-1.048649	1.662027
C	-3.832907	0.370779	0.674798
C	-2.948611	0.181169	1.961061
C	-3.725003	-0.003664	3.260031
H	-3.027183	-0.146972	4.090893
H	-4.334597	0.879939	3.477649
H	-4.378812	-0.876985	3.218378
C	-1.886943	1.274914	2.120140
H	-2.331368	2.237077	2.390671
H	-1.190125	0.979972	2.910082
H	-1.317570	1.398664	1.195128
C	-4.067376	1.818804	0.260985
H	-4.667908	1.845918	-0.653666
H	-4.609934	2.365196	1.040245
H	-3.127675	2.333147	0.060731
C	-5.160446	-0.392859	0.739248
H	-5.857286	0.060054	1.451141
H	-5.623767	-0.382456	-0.251496
H	-4.999557	-1.437503	1.021063
H	4.791292	2.577053	0.569906
H	2.827837	4.456712	-0.026059
C	-0.102324	-1.925145	-0.824595
C	-1.369770	-2.271853	-0.462967
C	3.810131	-0.043204	0.419656
C	4.653242	-0.528476	-0.591348
C	3.565285	-0.762317	1.601578
C	5.273259	-1.765933	-0.386397
C	4.203587	-1.995558	1.755313
C	5.060663	-2.512684	0.776520
H	5.925393	-2.160253	-1.161566
H	4.019435	-2.570684	2.659538
C	0.364235	3.285121	-0.552191
C	-0.301876	4.002589	0.456646
C	-0.155581	3.168065	-1.851331
C	-1.510473	4.622798	0.129733
C	-1.373992	3.800382	-2.124101
C	-2.062597	4.532915	-1.153243
H	-2.041392	5.175762	0.900951
H	-1.792727	3.715415	-3.123646
C	2.605285	-0.253731	2.646635
C	4.857214	0.238657	-1.874216
C	5.739507	-3.844474	0.985372
C	0.250144	4.081640	1.858880
C	0.551074	2.376383	-2.922016
C	-3.398922	5.162433	-1.458660
H	5.475532	-0.330610	-2.572703

Cu-Int2-α

H	3.899311	0.448644	-2.362952
H	5.346761	1.203921	-1.703631
H	6.499081	-3.781967	1.774508
H	5.020138	-4.611156	1.292629
H	6.234966	-4.192388	0.074459
H	2.745060	0.813669	2.845704
H	1.571117	-0.388898	2.306655
H	2.725649	-0.798820	3.586481
H	0.547807	3.093663	2.223309
H	1.136244	4.724020	1.916207
H	-0.495513	4.487913	2.546668
H	0.164900	2.631686	-3.912364
H	1.630720	2.556522	-2.915175
H	0.402148	1.300566	-2.766819
H	-3.513109	5.369204	-2.526947
H	-4.215944	4.490882	-1.165867
H	-3.537004	6.100772	-0.912102
C	-2.042881	-3.584151	-0.688289
C	-2.750559	-4.201831	0.363191
C	-2.057783	-4.222554	-1.943061
C	-3.416002	-5.413578	0.177269
H	-2.763215	-3.721192	1.338492
C	-2.723404	-5.434576	-2.131720
H	-1.553441	-3.747874	-2.779171
C	-3.403427	-6.039954	-1.071902
H	-3.945532	-5.870998	1.009691
H	-2.717823	-5.904091	-3.112565
H	-3.923215	-6.982877	-1.219357
C	0.843226	-2.912973	-1.494695
H	1.002938	-2.642771	-2.548739
H	1.833263	-2.864691	-1.024518
H	0.510932	-3.959802	-1.467837
Cu	0.700224	-0.218028	-0.438495

Cu-Int3-β

C	-0.834297	1.881116	0.426018
C	-0.031178	2.959683	0.177201
C	-0.462181	-1.875673	-0.243312
N	0.446644	-2.887611	-0.142148
N	-1.617255	-2.512631	-0.591308
C	-0.132380	-4.124086	-0.416261
H	0.434225	-5.040495	-0.375301
C	-1.437342	-3.885695	-0.702011
H	-2.244871	-4.549346	-0.967725
C	3.320090	1.669375	-1.034352
C	3.450569	3.221628	-1.262712
B	1.399082	2.726640	-0.370193
O	2.153397	1.582403	-0.177266
O	2.081088	3.675089	-1.108728
C	2.981623	0.889219	-2.309559
H	3.826421	0.853720	-3.004282
H	2.717803	-0.134441	-2.030683
H	2.122337	1.331514	-2.822342
C	4.501317	1.023737	-0.317890
H	4.308164	-0.042598	-0.188111
H	5.420583	1.136445	-0.902912
H	4.659869	1.460530	0.669718
C	3.950849	3.628001	-2.645179
H	4.004645	4.718786	-2.714405
H	4.953081	3.226924	-2.829331
H	3.282592	3.275801	-3.433339
C	4.268206	3.925034	-0.173111
H	5.335496	3.696712	-0.250918
H	4.137807	5.006138	-0.275258
H	3.921222	3.638725	0.823928
O	0.715304	0.995337	3.023499
C	2.135264	1.026669	2.996702
H	2.549333	0.457748	2.156538
H	2.527066	2.054641	2.940959
H	2.492319	0.584432	3.933732
H	0.395603	1.301502	2.153359
C	1.827799	-2.721596	0.222724
C	2.806419	-2.839716	-0.778161
C	2.154300	-2.482557	1.566403
C	4.146315	-2.718757	-0.398982
C	3.510272	-2.360176	1.892036
C	4.517032	-2.477245	0.929306
H	4.917615	-2.802319	-1.160861
H	3.781833	-2.168677	2.927072
C	-2.866141	-1.827064	-0.803595
C	-3.139845	-1.304011	-2.073883
C	-3.751449	-1.685471	0.276508
C	-4.356698	-0.637614	-2.254161
C	-4.953905	-1.015389	0.046478
C	-5.274119	-0.487357	-1.210030
H	-4.586415	-0.219772	-3.231140
H	-5.645438	-0.877958	0.873007
C	2.430351	-3.081302	-2.219892
C	5.966696	-2.284477	1.299529
C	1.093142	-2.339159	2.627158
C	-2.132635	-1.415952	-3.189974
C	-6.567729	0.262375	-1.406517
C	-3.380056	-2.179801	1.650849
H	1.619560	-2.419389	-2.539427
H	3.286931	-2.910990	-2.877117
H	2.085713	-4.109310	-2.383214
H	6.627893	-2.887204	0.668998
H	6.260413	-1.234654	1.170291
H	6.155176	-2.549288	2.344366
H	0.636613	-1.343043	2.591009
H	0.295114	-3.078406	2.506121
H	1.526512	-2.465200	3.623255
H	-1.248559	-0.805774	-2.968361

H	-1.784997	-2.446033	-3.325199
H	-2.554282	-1.069809	-4.137142
H	-6.744891	0.499388	-2.459613
H	-7.424350	-0.314153	-1.039577
H	-6.547814	1.205028	-0.847380
H	-4.210613	-2.055839	2.349792
H	-3.091647	-3.236602	1.645352
H	-2.526333	-1.612817	2.038410
C	-0.428785	4.415711	0.378927
H	-1.360596	4.527899	0.939322
H	0.357522	4.963134	0.914561
H	-0.551282	4.933904	-0.581832
C	-2.240863	2.051475	0.882702
C	-3.196826	2.752992	0.121536
C	-2.682562	1.438329	2.073477
C	-4.526411	2.846934	0.535726
H	-2.884892	3.220897	-0.808095
C	-4.005794	1.546644	2.497647
H	-1.964810	0.873439	2.662800
C	-4.939728	2.249017	1.728927
H	-5.241379	3.394611	-0.073878
H	-4.312374	1.072072	3.426762
H	-5.974327	2.324913	2.052702
Cu	-0.429481	0.016966	0.088237

Cu-TS2-β

C	-1.277130	1.044609	1.819197
C	-1.294884	2.204275	1.095030
C	0.214697	-1.420255	-0.900929
N	1.308185	-2.146905	-1.287685
N	-0.721662	-1.720454	-1.846179
C	1.049050	-2.886431	-2.437074
H	1.793324	-3.526547	-2.882756
C	-0.231924	-2.616801	-2.790651
H	-0.837829	-2.969251	-3.610067
C	2.301531	2.998361	0.210852
C	1.508905	3.779941	-0.903994
B	0.051243	2.723698	0.520915
O	1.261072	2.708715	1.182406
O	0.161376	3.256776	-0.751557
C	2.842243	1.646125	-0.261214
H	3.660642	1.759328	-0.977370
H	3.211353	1.087433	0.600982
H	2.049558	1.050203	-0.719965
C	3.403425	3.796274	0.899820
H	3.884522	3.175781	1.661735
H	4.169215	4.103364	0.179677
H	3.009189	4.686435	1.393230
C	1.969945	3.510531	-2.332689
H	1.340329	4.066396	-3.034046
H	3.005815	3.834616	-2.476676
H	1.897593	2.450787	-2.584752
C	1.424674	5.287542	-0.643800
H	2.389940	5.781827	-0.789692
H	0.702773	5.728563	-1.337286
H	1.079544	5.489748	0.374239
O	0.699296	-0.384894	2.730150
C	1.973295	0.084769	3.111974
H	2.105903	1.146270	2.860962
H	2.135704	-0.028070	4.198114
H	2.767345	-0.486324	2.607458
H	-0.106750	0.444494	2.443131
C	2.589722	-2.114368	-0.632448
C	3.657422	-1.478010	-1.293843
C	2.744470	-2.710032	0.628532
C	4.898918	-1.446108	-0.656440
C	4.011642	-2.647478	1.224284
C	5.095373	-2.021262	0.605303
H	5.730725	-0.951179	-1.152467
H	4.145454	-3.099652	2.203869
C	-2.052217	-1.171782	-1.890493
C	-2.249545	0.086410	-2.480035
C	-3.108319	-1.929226	-1.362853
C	-3.559545	0.574507	-2.540942
C	-4.401352	-1.410067	-1.472788
C	-4.645953	-0.160225	-2.051813
H	-3.733399	1.550199	-2.988455
H	-5.231951	-1.985072	-1.072220
C	3.479743	-0.833700	-2.647742
C	6.438112	-1.929365	1.288127
C	1.601958	-3.362933	1.363504
C	-1.094640	0.907482	-2.993990
C	-6.041086	0.413698	-2.088282
C	-2.845668	-3.227253	-0.643501
H	2.560014	-0.244279	-2.696801
H	4.319319	-0.171174	-2.872868
H	3.425760	-1.578344	-3.450496
H	7.259557	-2.105335	0.585407
H	6.588860	-0.931058	1.717878
H	6.526971	-2.653544	2.103138
H	1.069433	-2.611466	1.960153
H	0.879448	-3.820577	0.682198
H	1.973109	-4.136086	2.042811
H	-0.599221	1.434648	-2.171612
H	-0.343956	0.287759	-3.494246
H	-1.440918	1.663501	-3.704095
H	-6.199099	1.035388	-2.975268
H	-6.802022	-0.372721	-2.082474
H	-6.218823	1.048585	-1.211099
H	-3.780640	-3.700475	-0.334519
H	-2.288766	-3.939152	-1.262120
H	-2.247471	-3.044780	0.256742
C	-2.534333	3.007285	0.730074

H	-3.387819	2.779860	1.375083
H	-2.338736	4.085446	0.786507
H	-2.841386	2.806446	-0.305466
C	-2.506512	0.372386	2.307310
C	-3.615078	0.111554	1.476797
C	-2.550121	-0.122526	3.626857
C	-4.721906	-0.595028	1.947525
H	-3.587643	0.444452	0.445757
C	-3.665644	-0.808366	4.105271
H	-1.687936	0.032996	4.268770
C	-4.759272	-1.050940	3.267648
H	-5.557180	-0.789374	1.279215
H	-3.677553	-1.167356	5.131541
H	-5.623378	-1.596837	3.637193
Cu	-0.027060	-0.364109	0.666896

Cu-Int4-β

C	-2.145896	1.515494	1.285618
C	-1.926014	2.374495	0.258323
C	0.680124	-1.556210	-0.695739
N	1.865335	-2.050197	-1.169749
N	-0.226614	-1.917185	-1.647483
C	1.694175	-2.711338	-2.381127
H	2.517663	-3.173034	-2.901756
C	0.374175	-2.629636	-2.681637
H	-0.192840	-3.003185	-3.519304
C	1.756047	3.215191	0.472426
C	1.336481	3.896751	-0.884523
B	-0.472759	2.887387	0.085719
O	0.467149	2.948052	1.089441
O	0.024284	3.316523	-1.130539
C	2.446841	1.863002	0.280434
H	3.451037	1.973836	-0.137408
H	2.528919	1.359843	1.246852
H	1.861824	1.214035	-0.374429
C	2.571509	4.096112	1.412269
H	2.807899	3.539613	2.323983
H	3.515000	4.393584	0.942732
H	2.024681	4.995552	1.701047
C	2.235614	3.579728	-2.073995
H	1.861857	4.090281	-2.966825
H	3.259442	3.922372	-1.891675
H	2.258122	2.509325	-2.283939
C	1.126914	5.409759	-0.761333
H	2.074653	5.943204	-0.641959
H	0.636946	5.775203	-1.668432
H	0.481641	5.648569	0.089012
O	0.076719	-0.462363	2.780291
C	0.924793	0.399406	3.484621
H	0.843147	1.448336	3.150561
H	0.679466	0.387674	4.563196
H	1.995172	0.120677	3.411518
H	-1.287206	1.271178	1.908359
C	3.133801	-1.848205	-0.520580
C	4.023182	-0.909642	-1.070587
C	3.440033	-2.575055	0.641305
C	5.234300	-0.689497	-0.408819
C	4.664022	-2.315109	1.267157
C	5.568077	-1.373364	0.765303
H	5.929770	0.039689	-0.817632
H	4.914028	-2.864270	2.171678
C	-1.630761	-1.593448	-1.621371
C	-2.086659	-0.517783	-2.401164
C	-2.496422	-2.372322	-0.838659
C	-3.462579	-0.275270	-2.427251
C	-3.861032	-2.068042	-0.876179
C	-4.363474	-1.037591	-1.675198
H	-3.837016	0.544168	-3.035563
H	-4.542922	-2.643107	-0.256878
C	3.692420	-0.159111	-2.337465
C	6.859307	-1.078044	1.488497
C	2.485971	-3.590353	1.218195
C	-1.131264	0.397037	-3.127600
C	-5.843924	-0.753797	-1.737927
C	-1.973520	-3.472197	0.049895
H	2.648752	0.165403	-2.353727
H	4.327818	0.723731	-2.443019
H	3.847036	-0.783777	-3.225446
H	7.646487	-0.765463	0.795178
H	6.721953	-0.265816	2.213419
H	7.219827	-1.950205	2.042643
H	1.700663	-3.094659	1.801205
H	1.987738	-4.170728	0.435732
H	3.011072	-4.283217	1.881111
H	-0.665371	1.099441	-2.425071
H	-0.325759	-0.147831	-3.628914
H	-1.657024	0.990780	-3.880320
H	-6.283826	-1.165357	-2.655524
H	-6.371154	-1.193172	-0.887647
H	-6.044025	0.322468	-1.738122
H	-2.793867	-4.073003	0.450427
H	-1.287585	-4.137974	-0.484737
H	-1.419005	-3.050904	0.896950
C	-2.943512	2.786616	-0.781494
H	-3.507697	3.672467	-0.457161
H	-2.446662	3.060875	-1.717227
H	-3.667276	1.997148	-0.996248
C	-3.340451	0.737487	1.645693
C	-4.665643	1.089089	1.322793
C	-3.125551	-0.454880	2.371619
C	-5.730321	0.260602	1.679315
H	-4.872192	2.025767	0.819502
C	-4.190498	-1.286214	2.711118
H	-2.105187	-0.701522	2.661576
C	-5.498377	-0.936824	2.360771

H	-6.745831	0.554049	1.426431
H	-4.000774	-2.205239	3.259971
H	-6.330765	-1.581075	2.631916
Cu	0.378883	-0.831713	1.016077

Cu-Int3-α

C	-0.413536	-1.801099	0.687253
C	-1.697058	-1.890516	0.207747
C	2.167362	0.998147	0.168770
N	2.157299	2.342902	0.400877
N	3.482636	0.719884	-0.060102
C	3.437008	2.884651	0.325407
H	3.624121	3.933665	0.490499
C	4.275567	1.858284	0.033283
H	5.343571	1.827598	-0.113358
C	-2.254885	0.833259	-2.353542
C	-3.694683	0.228520	-2.199062
B	-2.232148	-0.846364	-0.803307
O	-1.589304	0.329932	-1.164770
O	-3.430714	-0.999007	-1.475517
C	-1.489547	0.269572	-3.556788
H	-1.880912	0.651207	-4.504844
H	-0.438393	0.561987	-3.472947
H	-1.533215	-0.823259	-3.574179
C	-2.199194	2.354977	-2.357704
H	-1.168226	2.688240	-2.495359
H	-2.799101	2.759234	-3.180166
H	-2.560620	2.773478	-1.418092
C	-4.396638	-0.114507	-3.508730
H	-5.384854	-0.535107	-3.299336
H	-4.532916	0.781439	-4.123707
H	-3.834560	-0.852397	-4.084456
C	-4.609110	1.081514	-1.311202
H	-4.927965	1.997569	-1.817691
H	-5.498914	0.498828	-1.056963
H	-4.107625	1.353833	-0.378556
O	-1.721703	0.009583	2.849979
C	-2.779749	0.699232	2.196609
H	-2.414838	1.332403	1.377677
H	-3.535914	0.007252	1.796689
H	-3.266454	1.340360	2.939130
H	-1.269171	-0.497501	2.150330
C	0.971537	3.100834	0.699473
C	0.445741	3.937147	-0.302572
C	0.376406	2.983197	1.962594
C	-0.718068	4.650386	-0.015165
C	-0.796841	3.714240	2.197212
C	-1.360813	4.542236	1.226169
H	-1.144090	5.293301	-0.781813
H	-1.278688	3.622357	3.167535
C	3.973785	-0.610138	-0.306536
C	4.118441	-1.047664	-1.630059
C	4.266623	-1.429105	0.794604
C	4.592792	-2.347101	-1.836641
C	4.736300	-2.719966	0.537762
C	4.907766	-3.194483	-0.768456
H	4.713408	-2.706178	-2.855848
H	4.968292	-3.370880	1.377242
C	1.107699	4.042637	-1.654486
C	-2.655775	5.274013	1.481122
C	0.922312	2.089719	3.047622
C	3.731296	-0.159390	-2.785315
C	5.386337	-4.604515	-1.015789
C	4.030264	-0.940114	2.200557
H	1.280926	3.053695	-2.091712
H	0.489408	4.620342	-2.346063
H	2.084485	4.535933	-1.592573
H	-3.491922	4.767275	0.982681
H	-2.889945	5.320076	2.548566
H	-2.622666	6.298243	1.094570
H	0.309902	1.183892	3.115767
H	1.958966	1.797269	2.870201
H	0.868122	2.593708	4.018331
H	2.647778	0.008037	-2.794725
H	4.205069	0.825946	-2.721134
H	4.011860	-0.611748	-3.739878
H	4.546861	-5.310712	-0.998443
H	5.872514	-4.700015	-1.991473
H	6.096869	-4.927965	-0.248411
H	4.364776	-1.676626	2.935143
H	4.552846	0.002717	2.397009
H	2.961045	-0.755501	2.362010
C	0.142413	-2.871432	1.613431
H	1.131600	-3.187396	1.259495
H	0.310996	-2.453661	2.616652
H	-0.483878	-3.765395	1.727212
C	-2.666428	-2.958471	0.620143
C	-3.083929	-3.062265	1.959238
C	-3.223211	-3.846798	-0.315036
C	-4.005729	-4.034237	2.352552
H	-2.686010	-2.358283	2.685557
C	-4.142024	-4.821303	0.075988
H	-2.931062	-3.762903	-1.357988
C	-4.536131	-4.921562	1.413196
H	-4.312696	-4.095091	3.393911
H	-4.553893	-5.502684	-0.664573
H	-5.253343	-5.679266	1.717875
Cu	0.788815	-0.337808	0.321789

Cu-TS2-α

C	-1.540062	0.453569	1.804476
C	-1.936850	1.391760	0.882543

C	0.919052	-1.796168	-0.290327
N	2.176962	-2.202322	-0.644819
N	0.107473	-2.746967	-0.837341
C	2.141855	-3.382654	-1.383770
H	3.039187	-3.859296	-1.744135
C	0.834552	-3.725679	-1.506011
H	0.357654	-4.561534	-1.992806
C	1.297100	2.658080	-0.580690
C	0.369580	3.908247	-0.780406
B	-0.812080	2.230382	0.232239
O	0.531575	1.889425	0.389559
O	-0.956116	3.342555	-0.566299
C	1.409281	1.787211	-1.833705
H	2.066658	2.234536	-2.584763
H	1.818610	0.821852	-1.534541
H	0.426881	1.616222	-2.282881
C	2.675824	2.935109	0.003472
H	3.217539	1.992651	0.124053
H	3.257666	3.577872	-0.665735
H	2.608895	3.414025	0.981673
C	0.416531	4.525824	-2.172776
H	-0.265402	5.379870	-2.223178
H	1.425611	4.883159	-2.403019
H	0.115265	3.810295	-2.940405
C	0.576049	4.980306	0.293457
H	1.525728	5.507046	0.160112
H	-0.235827	5.710262	0.230135
H	0.557945	4.540588	1.294731
O	0.764682	0.081289	3.010885
C	1.571702	1.189998	3.325644
H	1.216279	2.110652	2.836691
H	1.600907	1.380137	4.412684
H	2.611783	1.029488	2.996707
H	-0.269280	0.381672	2.520120
C	3.365140	-1.443566	-0.359071
C	4.016222	-0.791268	-1.424100
C	3.819141	-1.344340	0.965421
C	5.126658	0.000830	-1.125788
C	4.935213	-0.531929	1.209290
C	5.594013	0.152848	0.186005
H	5.631996	0.522569	-1.935330
H	5.293838	-0.437563	2.231177
C	-1.327057	-2.696099	-0.729101
C	-2.049142	-1.974074	-1.692628
C	-1.944259	-3.305541	0.369786
C	-3.432715	-1.883942	-1.536516
C	-3.334744	-3.191690	0.481111
C	-4.091739	-2.479178	-0.453181
H	-4.008141	-1.306741	-2.255419
H	-3.830371	-3.642379	1.337096
C	3.543037	-0.931995	-2.851401
C	6.771230	1.052407	0.473786
C	3.136517	-2.057266	2.104706
C	-1.338614	-1.263928	-2.816029
C	-5.579074	-2.293133	-0.280802
C	-1.121028	-3.985733	1.433385
H	2.453050	-0.895432	-2.928394
H	3.955046	-0.133537	-3.473950
H	3.861919	-1.885952	-3.287655
H	6.526511	2.098276	0.251812
H	7.074282	0.998260	1.523130
H	7.637586	0.787621	-0.143123
H	2.280474	-1.475912	2.471775
H	2.761215	-3.038657	1.798932
H	3.832410	-2.200702	2.936621
H	-0.700764	-0.467206	-2.417521
H	-0.688172	-1.939272	-3.382927
H	-2.052217	-0.814002	-3.510847
H	-5.807132	-1.248801	-0.036680
H	-6.123506	-2.534668	-1.200798
H	-5.975866	-2.920630	0.522515
H	-1.759170	-4.449394	2.189820
H	-0.467563	-4.759020	1.014818
H	-0.471942	-3.256641	1.933855
C	-2.513819	-0.451287	2.526386
H	-2.051161	-1.426811	2.710203
H	-2.741821	-0.038741	3.520198
H	-3.467277	-0.613247	2.009497
C	-3.342449	1.615754	0.433078
C	-4.412966	1.759233	1.333204
C	-3.635509	1.678189	-0.942633
C	-5.722778	1.929544	0.879796
H	-4.211741	1.747484	2.399686
C	-4.942098	1.842821	-1.400269
H	-2.821206	1.589623	-1.656285
C	-5.996082	1.964790	-0.489624
H	-6.530582	2.039372	1.599038
H	-5.139751	1.878576	-2.468903
H	-7.015432	2.095398	-0.842794
Cu	0.320046	-0.380576	0.831224

Cu-Int4-α

C	-2.144789	0.427524	1.945438
C	-2.495186	1.277654	0.946152
C	1.270759	-1.646082	-0.453836
N	2.444875	-1.713595	-1.152047
N	0.404971	-2.388586	-1.201344
C	2.311708	-2.489428	-2.299152
H	3.136035	-2.658953	-2.972937
C	1.024831	-2.917734	-2.328586
H	0.494288	-3.538452	-3.032805
C	0.664662	3.309975	0.496751
C	-0.167249	3.740190	-0.768358

B	-1.356563	2.233527	0.480625
O	-0.344242	2.654641	1.312822
O	-1.217450	2.731898	-0.800921
C	1.739421	2.266652	0.182335
H	2.566256	2.694620	-0.390937
H	2.138157	1.864864	1.116770
H	1.319098	1.429005	-0.377624
C	1.262123	4.459603	1.300185
H	1.801970	4.059432	2.163454
H	1.969864	5.032631	0.691754
H	0.490368	5.136600	1.670612
C	0.588766	3.690313	-2.090780
H	-0.074693	3.990532	-2.907682
H	1.441429	4.376997	-2.076985
H	0.953385	2.684799	-2.306403
C	-0.865921	5.093605	-0.601476
H	-0.152135	5.922178	-0.629218
H	-1.583576	5.227906	-1.415850
H	-1.416446	5.138157	0.342650
O	0.776279	-0.501866	3.023323
C	1.426928	0.597663	3.589506
H	0.985807	1.562007	3.279125
H	1.361458	0.562338	4.693698
H	2.508597	0.645420	3.350455
H	-1.143299	0.526889	2.361445
C	3.635951	-0.993449	-0.787760
C	3.966377	0.159253	-1.522678
C	4.416497	-1.442837	0.287468
C	5.104443	0.872846	-1.142318
C	5.546502	-0.689578	0.630314
C	5.903150	0.468621	-0.065075
H	5.369698	1.771518	-1.694653
H	6.159589	-1.020811	1.464569
C	-0.988032	-2.564179	-0.880222
C	-1.933576	-1.739820	-1.510804
C	-1.347119	-3.532924	0.067700
C	-3.277633	-1.919655	-1.173859
C	-2.704725	-3.670164	0.374800
C	-3.680594	-2.874917	-0.234452
H	-4.023353	-1.274718	-1.628401
H	-3.001597	-4.405831	1.118231
C	3.122983	0.617224	-2.687146
C	7.111684	1.281077	0.332291
C	4.050960	-2.678756	1.069880
C	-1.516906	-0.646548	-2.463717
C	-5.141284	-3.022342	0.115561
C	-0.299640	-4.360224	0.769163
H	2.054771	0.536682	-2.468872
H	3.343947	1.656703	-2.941644
H	3.310995	0.011818	-3.581918
H	6.813465	2.262532	0.720144
H	7.697449	0.781580	1.109094
H	7.770473	1.461529	-0.524741
H	3.242540	-2.461630	1.778188
H	3.697239	-3.482779	0.417078
H	4.908808	-3.046983	1.638845
H	-1.126364	0.217549	-1.913280
H	-0.735640	-0.972638	-3.157006
H	-2.369811	-0.300982	-3.052204
H	-5.626633	-2.043894	0.190951
H	-5.677816	-3.591830	-0.654097
H	-5.276493	-3.545441	1.066880
H	-0.759399	-5.161472	1.353189
H	0.404669	-4.811510	0.061885
H	0.289000	-3.735656	1.452647
C	-2.901440	-0.726690	2.528525
H	-2.220886	-1.579615	2.627254
H	-3.246001	-0.489517	3.545214
H	-3.764853	-1.033315	1.936112
C	-3.830281	1.284718	0.288979
C	-5.012731	1.084237	1.028895
C	-3.967011	1.523580	-1.093110
C	-6.264251	1.063894	0.411000
H	-4.951772	0.966065	2.105577
C	-5.217079	1.505403	-1.711720
H	-3.082385	1.726770	-1.682792
C	-6.374011	1.262462	-0.967132
H	-7.156155	0.904614	1.011593
H	-5.286842	1.684462	-2.781875
H	-7.347418	1.247791	-1.449625
Cu	0.993161	-0.927059	1.264358

Cu-Int5

C	1.841367	-1.110463	-0.742022
N	3.189771	-1.298523	-0.770974
N	1.344080	-2.129421	-1.499437
C	3.531743	-2.408010	-1.535877
H	4.557082	-2.709847	-1.678490
C	2.366120	-2.930442	-1.996701
H	2.166608	-3.783471	-2.624961
C	-2.325951	-1.119658	2.884576
C	-3.681438	-0.899124	2.123708
C	-1.747931	2.942640	-1.627950
C	-2.564236	3.667654	-0.498079
O	-3.272005	-0.082834	0.997027
O	-1.579476	0.069478	2.523519
O	-1.499389	1.664745	-1.034118
O	-1.940518	3.192949	0.692918
C	-4.692693	-0.071023	2.925793
H	-5.518000	0.214983	2.267174
H	-5.103233	-0.632745	3.770911
H	-4.232975	0.847145	3.302309
C	-4.340431	-2.170545	1.598094
H	-4.593033	-2.849957	2.419831

H	-5.267960	-1.916323	1.075825
H	-3.691526	-2.694616	0.895221
C	-1.523818	-2.311932	2.349135
H	-0.517086	-2.280006	2.777213
H	-1.980850	-3.270867	2.613924
H	-1.432806	-2.251865	1.262784
C	-2.435934	-1.192443	4.404402
H	-3.057631	-2.040109	4.713040
H	-1.440759	-1.327621	4.840411
H	-2.860896	-0.277578	4.821989
C	-4.036994	3.219407	-0.474702
H	-4.497718	3.584707	0.448850
H	-4.610715	3.610551	-1.322411
H	-4.099278	2.127964	-0.470376
C	-2.481705	5.194054	-0.527285
H	-2.868944	5.599644	-1.469357
H	-3.077642	5.614258	0.290037
H	-1.452428	5.535371	-0.396270
C	-2.511529	2.729988	-2.935179
H	-2.852270	3.681869	-3.358697
H	-1.862928	2.247856	-3.674606
H	-3.378580	2.084581	-2.780268
C	-0.406561	3.641059	-1.915729
H	0.209026	2.981623	-2.537440
H	-0.537019	4.588030	-2.451776
H	0.133425	3.822726	-0.984501
B	-1.394070	1.863242	0.414633
B	-2.097045	0.576146	1.332523
O	0.162614	1.927009	0.714585
C	0.552262	2.427677	1.991263
H	0.116775	3.422135	2.135427
H	0.212246	1.766782	2.797881
H	1.644923	2.504138	2.023654
C	-0.063537	-2.360914	-1.708524
C	-0.671000	-3.428855	-1.028361
C	-0.786629	-1.497784	-2.547938
C	-2.048118	-3.607705	-1.192750
C	-2.163315	-1.709461	-2.662204
C	-2.812431	-2.747234	-1.987809
H	-2.537088	-4.422367	-0.664613
H	-2.744022	-1.030831	-3.281478
C	4.120612	-0.444707	-0.079566
C	4.576261	-0.834552	1.188023
C	4.506734	0.764059	-0.680733
C	5.466902	0.017872	1.849050
C	5.394870	1.583108	0.021649
C	5.888561	1.225362	1.282481
H	5.831032	-0.265076	2.833501
H	5.704858	2.525131	-0.424286
C	0.119284	-4.332417	-0.113647
H	-0.547075	-4.986889	0.453426
H	0.818015	-4.969744	-0.667810
H	0.712227	-3.753431	0.601656
C	-0.138681	-0.341578	-3.262602
H	-0.151566	0.534854	-2.605821
H	0.894476	-0.555382	-3.549790
H	-0.700236	-0.086230	-4.165653
C	-4.310195	-2.893819	-2.084476
H	-4.655503	-3.841132	-1.660355
H	-4.804029	-2.081738	-1.538563
H	-4.652159	-2.841207	-3.123892
C	4.086824	-2.106383	1.833793
H	3.012112	-2.046766	2.041903
H	4.238829	-2.978831	1.189759
H	4.602954	-2.288662	2.779667
C	3.945934	1.179561	-2.017189
H	4.057735	0.390380	-2.768568
H	2.873093	1.391747	-1.934362
H	4.441971	2.079635	-2.388442
C	6.871722	2.118080	1.999314
H	6.602543	3.174205	1.894566
H	6.923233	1.885369	3.066863
H	7.881535	1.999872	1.586397
Cu	0.954608	0.360234	0.021809

Cu-TS3

C	0.985500	-1.829141	-0.442999
N	2.269412	-2.251567	-0.620432
N	0.233909	-2.777477	-1.066856
C	2.319020	-3.438025	-1.342479
H	3.251429	-3.921530	-1.586962
C	1.033061	-3.770809	-1.624817
H	0.614606	-4.603413	-2.167368
C	-2.474608	0.275323	2.711652
C	-3.396189	0.885865	1.599566
C	0.044115	3.365838	-1.844704
C	-0.656098	4.165548	-0.682702
O	-2.474575	1.021303	0.482196
O	-1.171488	0.814040	2.349492
O	-0.010148	2.020092	-1.354501
O	-0.260536	3.441789	0.493085
C	-3.895325	2.294361	1.939293
H	-4.364000	2.729530	1.052692
H	-4.635285	2.277465	2.745703
H	-3.064865	2.941830	2.232025
C	-4.555554	-0.002985	1.163825
H	-5.246172	-0.180210	1.995549
H	-5.111224	0.490140	0.360185
H	-4.201146	-0.962606	0.787101
C	-2.355715	-1.251631	2.621540
H	-1.536212	-1.580261	3.267709
H	-3.273297	-1.752478	2.945928
H	-2.128981	-1.561850	1.599056

C	-2.801320	0.703267	4.137382
H	-3.811177	0.385140	4.418491
H	-2.095083	0.239803	4.833351
H	-2.728930	1.785564	4.258667
C	-2.187844	4.125535	-0.786159
H	-2.613269	4.528245	0.138007
H	-2.559174	4.727688	-1.622669
H	-2.535840	3.096926	-0.895815
C	-0.183199	5.612184	-0.534140
H	-0.372020	6.187287	-1.448179
H	-0.722954	6.096367	0.286503
H	0.883246	5.660094	-0.304121
C	-0.682617	3.415820	-3.187531
H	-0.762591	4.443343	-3.559964
H	-0.127272	2.830316	-3.928428
H	-1.685212	2.990259	-3.110670
C	1.516054	3.768482	-2.035636
H	1.989165	3.053831	-2.716378
H	1.618322	4.770441	-2.466641
H	2.051978	3.726332	-1.085537
B	0.118247	2.091874	0.096004
B	-1.187802	1.104443	0.985481
O	1.530303	1.758556	0.479654
C	1.924341	2.054975	1.810633
H	1.947381	3.139669	1.977730
H	1.240504	1.612365	2.549532
H	2.927981	1.647054	1.973265
C	-1.203133	-2.739713	-1.156572
C	-1.945985	-3.570477	-0.303381
C	-1.809949	-1.875443	-2.082673
C	-3.339648	-3.541598	-0.413438
C	-3.206859	-1.885756	-2.153187
C	-3.986193	-2.711360	-1.336026
H	-3.931471	-4.176763	0.240976
H	-3.697347	-1.223225	-2.862068
C	3.411285	-1.524844	-0.125937
C	4.023116	-1.967229	1.059493
C	3.839472	-0.379551	-0.812294
C	5.104569	-1.232393	1.548165
C	4.924194	0.325891	-0.276280
C	5.567967	-0.082359	0.894400
H	5.590420	-1.556386	2.465613
H	5.264046	1.222835	-0.787585
C	-1.264229	-4.437535	0.725855
H	-1.999146	-4.941597	1.358543
H	-0.635141	-5.206477	0.263590
H	-0.612462	-3.839706	1.372328
C	-1.003150	-0.936429	-2.942742
H	-0.725246	-0.033948	-2.382720
H	-0.077679	-1.400618	-3.296976
H	-1.581709	-0.618950	-3.814935
C	-5.492446	-2.666187	-1.418786

H	-5.948992	-3.562714	-0.988762
H	-5.885179	-1.800243	-0.871783
H	-5.833181	-2.574515	-2.455205
C	3.506852	-3.179506	1.794377
H	2.452893	-3.054943	2.066737
H	3.572089	-4.087359	1.184268
H	4.075049	-3.350327	2.712135
C	3.139402	0.114170	-2.052198
H	2.864117	-0.708267	-2.719925
H	2.222911	0.653509	-1.785447
H	3.782102	0.802791	-2.607395
C	6.744801	0.684587	1.447238
H	6.867810	1.649470	0.947434
H	6.627582	0.872844	2.520066
H	7.678027	0.121902	1.319798
Cu	0.483950	-0.131808	0.241527
